# HiPSC-Derived Hepatocyte-like Cells Can Be Used as a Model for Transcriptomics-Based Study of Chemical Toxicity

**DOI:** 10.3390/toxics10010001

**Published:** 2021-12-21

**Authors:** Sreya Ghosh, Jonathan De Smedt, Tine Tricot, Susana Proença, Manoj Kumar, Fatemeharefeh Nami, Thomas Vanwelden, Niels Vidal, Paul Jennings, Nynke I. Kramer, Catherine M. Verfaillie

**Affiliations:** 1 Department of Development and Regeneration, Stem Cell Institute, KU Leuven, 3000 Leuven, Belgium; jonathan.desmedt@kuleuven.be (J.D.S.); tine.tricot@kuleuven.be (T.T.); manoj.kumar@kuleuven.be (M.K.); fatemeharefeh.nami@kuleuven.be (F.N.); thomas.vanwelden@remynd.com (T.V.); niels.vidal@kuleuven.be (N.V.); 2 Institute for Risk Assessment Sciences, Utrecht University, P.O. Box 80177, 3508 TD Utrecht, The Netherlands; s.proenca@uu.nl (S.P.); N.I.Kramer@uu.nl (N.I.K.); 3 ReMYND NV, Bio-Incubator Leuven, Gaston Geenslaan 1, 3001 Leuven, Belgium; 4 Division of Molecular and Computational Toxicology, Department of Chemistry and Pharmaceutical Sciences, Amsterdam Institute for Molecules, Medicines and Systems, Vrije Universiteit Amsterdam, De Boelelaan 1108, 1081 HZ Amsterdam, The Netherlands; p.jennings@vu.nl; 5 Division of Toxicology, Wageningen University, P.O. Box 8000, 6700 EA Wageningen, The Netherlands

**Keywords:** mechanistic toxicity, transcriptomics, in vitro toxicology, stem cell derived, hepatocytes, ER stress

## Abstract

Traditional toxicity risk assessment approaches have until recently focussed mainly on histochemical readouts for cell death. Modern toxicology methods attempt to deduce a mechanistic understanding of pathways involved in the development of toxicity, by using transcriptomics and other big data-driven methods such as high-content screening. Here, we used a recently described optimised method to differentiate human induced pluripotent stem cells (hiPSCs) to hepatocyte-like cells (HLCs), to assess their potential to classify hepatotoxic and non-hepatotoxic chemicals and their use in mechanistic toxicity studies. The iPSC-HLCs could accurately classify chemicals causing acute hepatocellular injury, and the transcriptomics data on treated HLCs obtained by TempO-Seq technology linked the cytotoxicity to cellular stress pathways, including oxidative stress and unfolded protein response (UPR). Induction of these stress pathways in response to amiodarone, diclofenac, and ibuprofen, was demonstrated to be concentration and time dependent. The transcriptomics data on diclofenac-treated HLCs were found to be more sensitive in detecting differentially expressed genes in response to treatment, as compared to existing datasets of other diclofenac-treated in vitro hepatocyte models. Hence iPSC-HLCs generated by transcription factor overexpression and in metabolically optimised medium appear suitable for chemical toxicity detection as well as mechanistic toxicity studies.

## 1. Introduction

Chemical safety assessments are crucial to the research and development pipeline of pharmaceutical, nanomaterial, agrochemical, and cosmetic industries. Most late-stage pre-clinical studies and industrial risk assessment approaches depend upon the use of whole-animal models, such as rodents. However, differences in phase I and II metabolising enzymes and transporters [1,2] negatively impact risk assessment accuracy and extrapolation to chemical responses in humans. This is a major reason for the high cost and poor efficiency of drug development [3]. In addition, the ethical concerns related to using animals for chemical safety evaluation have led to the adoption of directive 2010/63/EU by the European Union, which calls for the use of the reduction, refinement and replacement (3Rs) principle in chemical safety evaluations. Thus, animal-free chemical safety evaluation models need to be developed, that sufficiently predict human toxicological responses and can be used by industry and regulatory bodies.

With regards to identifying hepatotoxicants, one can use human in vitro models, which currently include primary human hepatocytes (PHHs) or stable cell lines of hepatic origin, such as the HepG2 [4] and HepaRG models [5], transiently immortalised PHHs [6] or spheroids [7]. While PHHs remain the gold standard of in vitro models​, they are scarce as they are usually obtained from cadavers, transplants, or biopsies. Additionally, donor-specific polymorphisms lead to differences in drug metabolism and clearance rates [8], which may contribute to batch-to-batch differences observed for PHHs. Moreover, PHHs rapidly lose mature hepatic functions in two-dimensional (2D) culture, rendering them unsuitable for long-term exposure studies [9]. Transformed cell line models also do not have biotransformation capacity comparable to that of freshly isolated PHHs [10].

With the discovery by Yamanaka and colleagues in 2006 [11] that nucleated somatic cells can be reprogrammed to induced pluripotent stem cells (iPSCs), an inexhaustible human cell population can now be created that theoretically can differentiate into any human cell type. As part of the in3 project (Marie Sklodowska-Curie Action-Innovative Training Network 2017–2020 grant no. 721975, www.estiv.org/in3, accessed on 28 November 2021), iPSCs were differentiated to different cells representing lung, kidney, liver, and brain, for toxicity risk assessment studies, and the work on hepatocytic progeny is presented here. Numerous protocols have been described for the targeted differentiation of hiPSCs to cells with hepatocyte function, termed hepatocyte-like-cells (HLCs) [12,13,14]. We recently described that overexpression of three transcription factors (TFs; termed 3x) combined with the use of a metabolically improved medium (consisting of high concentrations of amino acids and glycine, termed AAGLY) generates transcriptionally more mature HLCs with improved cytochrome functional activity [15]. These cells can also be maintained in culture without loss of function for at least 50 days.

Until recently, the study of toxicology used mostly dose-response studies to identify and characterise toxicity risk. However, insights in toxicity mechanisms may improve predictions of toxicity risk at lower doses and shorter exposure times. Transcriptomics studies measure thousands of transcripts simultaneously and hence provide the means to understand toxicity at a pathway-level, offering insights into early-time point events that could also be used to predict toxicity caused by structurally similar chemicals. A limited number of studies have been published that address mechanistic toxicity using transcriptomics methods [7,16] and/or high-content imaging [17,18].

Here we used day 40 3x-genome and AAGLY-metabolically engineered (3x-AAGLY) iPSC-HLC progeny to evaluate their potential as a model for traditional dose-response toxicity studies and their ability to identify adverse effects mechanisms. For the purpose of in vitro tissue-specific toxicity risk assessment, ten chemicals linked to toxicity in different organs were chosen by the in3 network. Four other chemicals were chosen for this study, out of which two have been classified as highly likely to cause clinically apparent hepatocellular injury. The 3x-AAGLY-HLCs correctly categorised amiodarone, paraquat, diclofenac and clozapine as acutely cytotoxic to hepatocytes. Transcriptomics data (using the TempO-Seq method) on cells treated with amiodarone, paraquat, clozapine, diclofenac, gentamicin, olanzapine, and ibuprofen at or lower than an IC10 concentrations, were used to gain mechanistic insights in how these chemicals may cause hepatotoxicity. Additionally, we examined whether genes involved in the ER stress and UPR pathways were induced increasingly over time upon chemical treatment. Finally, we compared our transcriptome dataset with available datasets of other in vitro liver toxicity models to benchmark the sensitivity of 3x-AAGLY-HLCs to diclofenac.

## 2. Materials and Methods

Pluripotent stem cells: The SBAD2 iPSC line was derived by the Innovative Medicines Initiative-funded StemBANCC consortium (grant agreement no 115439, http://stembancc.org, accessed on 28 November 2021) from human adult dermal fibroblast cells. The human embryonic stem cell (hESC) line H9 (WA09, WiCell research institute, Madison, WI, USA) with three transcription factors (H9-3x) was generated as described in [15]. The use of hESCs and iPSCs for this study was approved by the “Commissie Medische Ethiek”, UZ KU Leuven/Onderzoek UZ Gasthuisberg, Herestraat 49, B 3000 Leuven, file number S-52426.

Primary human hepatocytes: As positive control for hepatocyte marker gene expression and function, hepatocytes were obtained from two cadaveric donors F125 (male, 62 years of age) and F133 (male, 57 years of age) from the Ministry of Health-accredited tissue banks at the Cliniques Universitaires St Luc, Brussels. This post-mortem collected tissue was obtained under the Belgian legislation on organ and tissue donation. The organs were collected within the ‘opting out system’, which uses the principle that if the deceased donor or her/his representative have not opposed organ donation, including for research purposes, organs can be obtained post-mortem. Cells from the donors were used for gene expression assessment and functional characterisation. The PHHs were thawed and plated on collagen I-coated plates using Primary Hepatocyte Thawing and Plating Supplements (Gibco Cat #CM3000). 

Commercially available PHHs pooled from different donors (LiverPool™ Cryoplateable Hepatocytes, Sigma-Aldrich X008001-P) were thawed and plated at 80% viability in between collagen (Rat tail collagen I, Corning) and growth-factor reduced matrigel layers [19,20]. They were cultured in Williams E medium for 4 days, followed by 24 h in Williams E with 2% FBS and 1% DMSO, followed by lysis for TempO-Seq.

HepG2 cell line: HepG2 cells were grown in T25 flasks until confluent in Dulbecco’s modified Eagle’s medium (DMEM) supplemented with 10% fetal bovine serum (FBS), and 100 units/mL penicillin, 100 μg/mL streptomycin, and then plated in 24-well plates at 100,000 cells/well. When confluent, they were used as a control for the characterization of HLCs.

HepaRG cell line: Human hepatoma HepaRG cells were cultured in Williams’ E medium without phenol red and L-glutamine (ThermoFisher A1217-01, Waltham, MA, USA) supplemented with 10% FBS (Gibco 10270-106), 2 mM glutamine (Gibco 25030-024), 100 units/mL penicillin 100 μg/mL streptomycin (Gibco 15140-122), 872 nM insulin (Sigma-Aldrich I6634, St. Louis, MO, USA), and 50 μM hydrocortisone hemisuccinate (Santa Cruz SC-250130, Santa Cruz, CA, USA). The cells were passaged every ten days by washing with PBS and trypsinization. For TempO-Seq, a T-75 flask was maintained seven days in culture, followed by another week in medium containing 1% DMSO (Sigma-Aldrich). The cells were trypsinized and 250 μL of a 130,000 cells/mL cell suspension was added to each well of a 48-well plate (Greiner). After two days in culture the medium was changed to 2% FBS and 1%DMSO and cells maintained with this medium composition till day six. The cells were lysed using 1X TempO-Seq buffer and two wells were pooled together for each sample for sequencing.

Generation of the SBAD2-3x iPSC line: As described before [21], a flippase recombinase target (FRT)-flanked donor cassette was integrated using zinc finger nucleases into the AAVS1 locus of the SBAD2 iPSC line, to generate the master cell line for recombinase mediated cassette exchange (RMCE). RMCE was performed by nucleofection of the master FRT-iPSC line with a FLPe-expressing vector and a donor vector containing Hepatocyte Nuclear Factor 1 Alpha (HNF1A), Forkhead Box A3 (FOXA3) and Prospero Homeobox Protein (PROX1), linked by 2A sequences and under control of a TetON promoter (SBAD2-3x line). A schematic representation of the genome engineering of the SBAD2-3x line is shown in Figure S1. Characterisation of the lines thus generated is described in Supplementary Methods, Table S1 and Figures S2–S5.

Cell culture and differentiation to HLCs: The iPSC lines were maintained on Matrigel (Corning 354277, Corning, NY, USA)-coated plates in Essential 8 Flex (ThermoFisher A2858501). For differentiation, they were seeded on growth-factor reduced matrigel coated plates at a density of ~8.75 × 10^4^ cells/cm^2^ and maintained in mTeSR medium (Stem Cell Technologies 85850) for 2–3 days. The differentiation was done as described in [15]. Briefly, the cells were maintained in liver differentiation medium (LDM) with sequential growth factor cocktails (as described in Figure S6). Between day 0 and day 12, 0.6% dimethyl sulphoxide (DMSO) was added, between day 12 and 14, 2% DMSO, and from day 14 onwards DMSO was omitted. From day 4 onwards, doxycycline 5 μg/mL was added until the end of culture. From day 12 onwards LDM was supplemented with 8 mL MEM amino acids and 16 mL MEM non-essential amino acids solution per 100 mL, and further supplemented with 20 g/L glycine from day 14 onwards. The media compositions are listed in Table S2. All growth factors were purchased from Peprotech (East Windsor, NJ, USA).

Quantitative real time polymerase chain reaction: Cells were lysed and RNA was extracted with the GenElute™ Mammalian RNA Extraction Kit (Sigma-Aldrich RTN70) and reverse transcribed into cDNA using SuperScript^®^ III First-Strand Synthesis SuperMix (Invitrogen™ 18080400), according to the manufacturer’s protocol. qRT-PCR reactions were prepared using the Platinum^®^ SYBR^®^ Green RT-qPCR SuperMix-UDG kit (ThermoFisher Scientific 11733046). All primer sequences used are listed in Table S3.

Immunostaining: Immunostaining of iPSC and iPSC-progeny was performed as described before [15]. The cells were imaged on an Axioimager Z1 microscope (Carl Zeiss, Jena, Germany). The antibodies used are listed in Table S4. Staining was quantified based on intensity in segmented cells (using the EBImage R package) and expressed as percentage of cells stained with each antibody. 

SBAD2-3x-AAGLY HLC functional assays: CYP3A4-dependent activity was measured by the metabolization of the fluorometric probe 7-Benzyloxy-4-trifluoromethylcoumarin (BFC; Sigma-Aldrich B5057), added for 4 h to the day 40 SBAD2-3x progeny as described [22]. Albumin secretion by day 40 SBAD2-3x progeny was quantified using the human albumin ELISA quantification kit (Bethyl E88–129) according to manufacturer’s instructions.

Chemical toxicity dose-response studies: Day 40 SBAD2-3x-AAGLY HLCs were treated with chemicals at the concentrations listed in Table S5a. The LiverTox DILI likelihood scores, type of liver injury, and cytotoxic IC50 values (of the chemicals causing acute hepatocellular injury) are listed in Table S5b. The treatment concentrations were chosen based on literature [23,24,25,26,27,28,29,30]. After 48 h of treatment, medium was collected for lactate dehydrogenase (LDH) assay as a measure of cytotoxicity. We used the cytotoxicity detection kit (Roche 11644793001) and followed the manufacturer’s instructions. Cytotoxicity was calculated using the following formula, where Abs denotes the absorbance at 490 nm, PC is positive control (cells killed with 0.1% triton-x-100), and NC is the negative control (untreated cells):

(1) $$\mathrm{Cytotoxicity}\;\left(\%\right)=100\times \frac{Abs_{sample}-Abs_{NC}}{Abs_{PC}-Abs_{NC}}$$

We used the drc R package to fit a four-parameter logistic regression curve for each chemical treatment.

TempO-Seq: Two independent differentiations of SBAD2-3x-AAGLY-HLCs were treated with amiodarone, paraquat, gentamicin, diclofenac, ibuprofen, clozapine, and olanzapine on day 40 of differentiation in 96-well plates (Greiner) at the concentrations and times listed in Table S6. After incubation, the cells were lysed using 1X TempO-Seq Lysis Buffer, and frozen lysates shipped to Bioclavis (Glasgow, Scotland) for sequencing. The probeset panel (Table S7) was based on the S1500+ gene panel identified by [31] and supplemented further as described in [32,33]. These genes were selected for their involvement in all major cellular stress pathways, as well as cell-type specific marker genes, to provide information on the cell-type and cell state upon chemical treatment.

Additionally, we compared the expression of genes involved in xenobiotic metabolism and other mature hepatocyte markers of HLCs to differentiated HepaRG cells and PHHs. TempO-Seq data from sandwich cultured PHH and 2D cultures of differentiated HepaRG (each cultured for 6 days after seeding) were used, and the log2CPMs of expression values were plotted. 

DESeq2: The raw counts matrix (Table S8) was obtained from BioSpyder. Subsequently, DEseq2 [34] was used to fit a generalised linear model of the form Expression=β×Concentration+ε for each chemical and for each gene separately. Differentially expressed genes were called using the Wald test. *p*-values were adjusted with the Benjamini-Hochberg correction for multiple-test comparisons. Prior to generating heatmap visualisations we converted the log2 CPM values of the top 20 most differentially expressed genes to Z-scores for each chemical.

Enrichment of pathways in DEGs: The gene-pathway annotations made for chemical stress response pathways by Wellens et al. [33,35] was used to link the concentration of chemical treatments to the elicitation of stress responses. A Z-score was calculated using the formula from Kutmon et al. [36]:

(2) $$Z-\mathrm{score}=\frac{\left(r-n\frac{R}{N}\right)}{\sqrt{n\left(\frac{R}{N}\right)\left(1-\frac{R}{N}\right)\left(1-\frac{n-1}{N-1}\right)}}$$

where N is the total number of TempO-Seq probes, R is the number of differentially expressed probes (*p* < 0.05), r is differentially expressed probes in a particular stress pathway, and n is the total number of TempO-Seq probes that were associated with the specific pathway. A z-score of less than 1.96 was applied as a threshold, and the enrichment Z scores thus calculated indicates the extent to which a given pathway is over- or under-represented in the differentially expressed genes in response to chemical treatment.

WGCNA, GO enrichment, and GSVA: The WGCNA R package [37] was used to generate signed co-expression networks for both the TempO-Seq data (with read counts converted to log2 counts-per-million (CPM)) and the human samples of the Open Toxicogenomics Project-Genomics Assisted Toxicity Evaluation Systems (TG-GATES) dataset [38]. Additionally, Gene Ontology (GO) enrichment analysis was performed on each module using the topGO R package. Enriched GO terms were used to manually annotate each co-expression module. Furthermore, Gene Set Variant Analysis (GSVA) was applied (using the GSVA R package) to summarize the gene expression values in each co-expression module and to assess module activities for each chemical [39].

RT-qPCR analysis of time-dependent differential expression of stress pathway genes: To further investigate temporally distinct induction of stress pathway genes, we selected the most cluster-central (kME > 0.65) genes from the WGCNA analysis on TempO-Seq (UPR module) and TG-GATES (UPR/ER stress, RNA metabolism, and general/xenobiotic metabolism modules). These were narrowed down to 10 genes based on literature evidence [40,41,42,43,44,45] of toxicity (Table S9), and differential expression in TempO-Seq in at least one of the three chemicals in common between the two datasets (i.e., amiodarone, diclofenac, or ibuprofen). Day 40 SBAD2-3x-AAGLY-HLCs were treated with diclofenac at 0, 12.5, 25, 50, and 100 μM, ibuprofen at 0, 25, 50, 100, and 200 μM, or amiodarone at 0, 2.5, 5, 10, and 20 μM, respectively. Cell lysates were collected 2, 4, 6, 8, and 24 h after exposure for each condition and expression of the selected genes was determined using RT-qPCR. In addition, to assess if the IRE1 arm of the endoplasmic reticulum (ER) stress and subsequent UPR pathway was also induced, we evaluated the expression of *sXBP1* (spliced *XBP1*) mRNA with the RT-qPCR primers described in [46].

Benchmarking: We manually assessed the literature for hepatocyte models with transcriptomics data in response to chemical treatment. We compared the SBAD2-3x-AAGLY-HLCs TempO-Seq data with four other studies GSE51952, GSE147866 [47], GSE40117 [48], and E-MTAB-798 [38,49], as all these studies contained responses to diclofenac. Gene symbols of rat samples (in GSE40117) were converted to their respective human gene ortholog symbols using the biomaRt R package. Log2 fold changes of gene expression following diclofenac treatment with its respective controls were determined for each of the models. PHH samples from E-MTAB-798, i.e., the TG-GATES dataset (exposed for 24 h at the highest concentration of diclofenac) were used as ‘gold standard’ to compare with each of the models. Genes were removed from analysis if they were not present in each of the datasets and if they were not differentially expressed in the ‘gold standard’ samples. We further defined a similarity metric for each gene’s induction for each of the models upon diclofenac treatment, Equation (3).

(3) $$S_{i}=\frac{\log_{2}{\mathrm{FC}}_{\mathrm{model}}}{\log_{2}{\mathrm{FC}}_{\mathrm{gold}}}$$

$S_{i}$ is the similarity of the i-th gene, ${\mathrm{FC}}_{\mathrm{model}}$ the fold change of the gene in the respective model upon diclofenac treatment, and ${\mathrm{FC}}_{\mathrm{gold}}$ the fold change of the gene in the gold standard samples upon diclofenac treatment. If $S_{i\;}$= 1, then the induction of gene i upon diclofenac treatment is equal in the respective model and the gold standard samples. We then calculated a recovery curve for each model, counting how many of the significantly differentially expressed genes in the gold standard samples were recovered under a range of thresholds applied on $S_{i}$.

## 3. Results

### 3.1. SBAD2-3x-AAGLY-HLCs Express Hepatocyte Markers, Produce Albumin and Have CYP3A4 Activity

**Figure 1:**
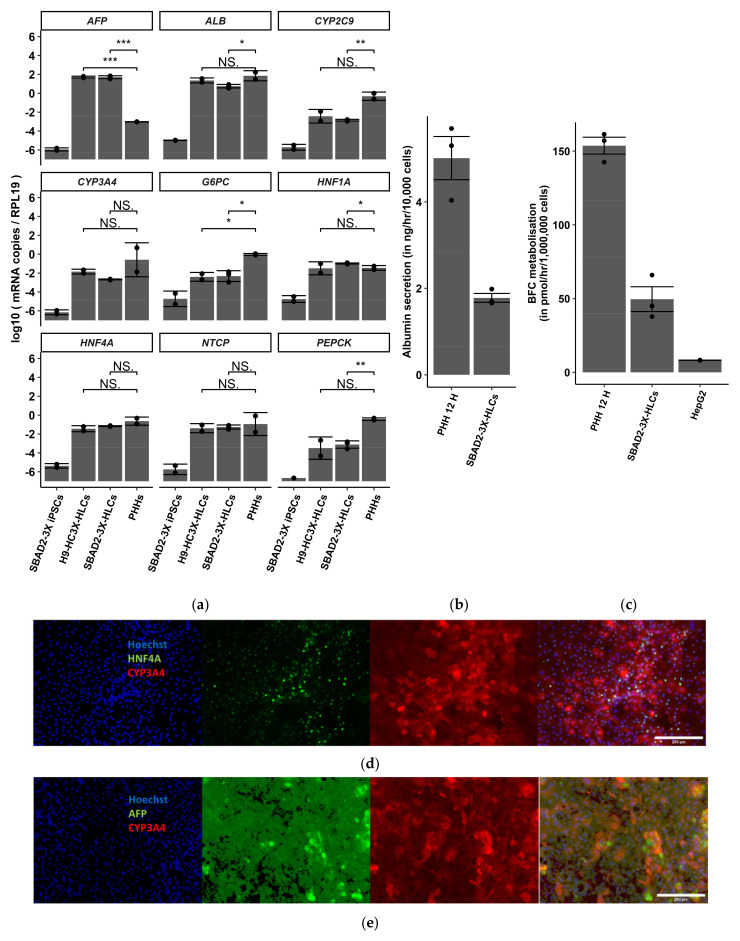
SBAD2-3x-AAGLY HLCs express hepatocyte markers, produce albumin, and have CYP3A4 activity. (**a**) Expression of hepatocyte markers in differentiated day 40 SBAD2-3x-AAGLY HLCs, H9-ESC-3x-AAGLY-HLCs and non-cultured PHH. (*n* = 3 biological replicate differentiations and *n* = 2 PHH donors). The significance was compared to that in undifferentiated SBAD2-3x iPSCs by unpaired 2-tailed Student’s *t*-test. (* *p* < 0.05, ** *p* < 0.01, *** *p* < 0.001, NS: Not significant). (**b**) Comparison of albumin secretion by SBAD2-3x-AAGLY HLCs and 12 h plated PHH by Albumin ELISA. (*n* = 2 PHH donors, *n* = 3 HLCs). The values for albumin secretion for 12 h PHH were used from Boon et al., Nature Communications, 2020, Figure 1d. (**c**) BFC metabolization of SBAD2-3x-AAGLY HLCs was compared to that of thawed cryopreserved PHH and the HepG2 cell line. *n* = 2 PHH donors, (*n* = 3 HLC differentiations, *n* = 2 HepG2 cells). (**d**) Immunofluorescence staining for CYP3A4 and HNF4A in day 40 differentiated SBAD2-3X HLCs. (**e**) Immunofluorescence staining for CYP3A4 and AFP in day 40 differentiated SBAD2-3X HLCs. Scale bar = 200 μm. (Representative images of *n =* 2 independent differentiations). Scale bar: 200 μm.

SBAD2-3x cells were differentiated using the protocol described in [15] for 40 days (Figure S6), and expression of characteristic hepatocyte gene markers was demonstrated by RT-qPCR and immunostaining. The cells displayed expression of *ALB*, *AFP*, *CYP3A4*, *CYP2C9*, *G6PC*, *NTCP*, *PEPCK*, and *HNF4A.* (relative to *RPL19*), similar to the expression levels in H9-HC3x-AAGLY HLCs (Figure 1a). The cells secreted ±40% as much albumin as secreted by 12-h plated PHHs and the CYP3A4 enzymatic activity of SBAD2-3x-AAGLY-HLCs was around 3-fold lower than that of 12-h plated PHHs (Figure 1b,c). Immunostaining confirmed the expression of HNF4A, AFP, and CYP3A4 proteins in day 40 progeny. Consistent with the CYP3A4 function, more SBAD2-3x-AAGLY-HLCs (Figure 1d,e) stained positive for CYP3A4 than HepG2 cells (Figure S7). Approximately 83.4 ± 15.5% HLCs and 25.76 ± 13.55% HepG2 cells were CYP3A4-positive, while 80.8 ± 17.4% HLCs and 99.9% HepG2 cells were HNF4A-positive. Both cell types also showed high expression of AFP (81.5 ± 15% HLCs and 86.8 ± 15% HepG2 cells). Persistent expression of AFP, lower albumin secretion and CYP3A4 activity indicate that the SBAD2-3x-AAGLY-HLCs are not as mature as PHHs. SBAD2-3x-AAGLY-HLCs showed lower BFC metabolization than HLC progeny from the 3x-hESC line we described previously [15], possibly due to donor-specific differences.

### 3.2. SBAD2-3x-AAGLY HLCs Cells Accurately Classify Chemicals Causing Acute Hepatocellular Injury

**Figure 2:**
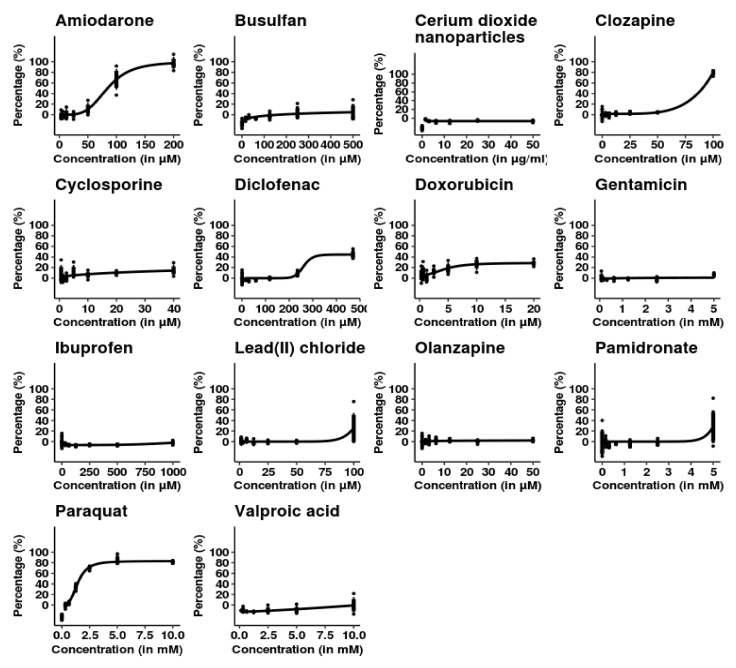
SBAD2-3x-AAGLY HLCs cells accurately classify hepatotoxic and non-hepatotoxic chemicals.

Day 40 SBAD2-3x-AAGLY HLCs were treated with increasing concentrations of 14 chemicals (Table S5a,b) for 48 h (Figure 2). No toxicity was observed following treatment with chemicals that are not known to cause acute toxicity in hepatocytes, i.e., gentamicin, lead (II) chloride, cerium dioxide nanoparticles, busulfan, doxorubicin, cyclosporine A, pamidronate, ibuprofen, and olanzapine. Dose-dependent cytotoxicity was observed for known acutely hepatotoxic chemicals including paraquat, amiodarone, diclofenac, and clozapine, but not for valproic acid. Valproic acid is known to have an acute inhibitory effect on mitochondrial respiration and ultimately steatosis, but only causes cytotoxicity at high concentrations and longer-term treatment. Hence, absence of acute toxicity observed here is in accordance with clinical observations [50].

Day 40 SBAD2-3x-AAGLY HLCs were treated with 14 chemicals: 11 prescription drugs, 1 industrial chemical at nanoparticle size, 1 heavy metal salt and 1 pesticide, at concentrations listed in Table S5. HLCs showed a dose-dependent increase in LDH activity (in black) upon treatment with amiodarone, paraquat, diclofenac, and clozapine. The y-axis indicates percentage LDH release with respect to control. No change in LDH levels were observed in response to treatment with the other 10 chemicals. (*n* = 3 independent differentiations and chemical treatments; smooth lines represent four-parameter logistic regression fits).

### 3.3. Cellular Stress Pathway Genes Are Highly Differentially Expressed in SBAD2-3x-AAGLY HLCs upon Chemical Treatment

**Figure 3:**
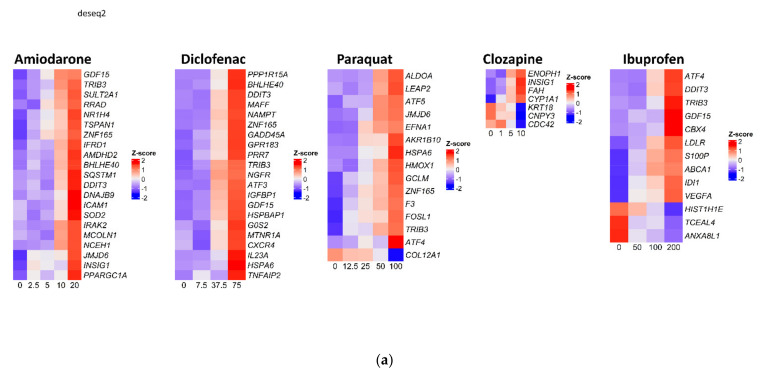

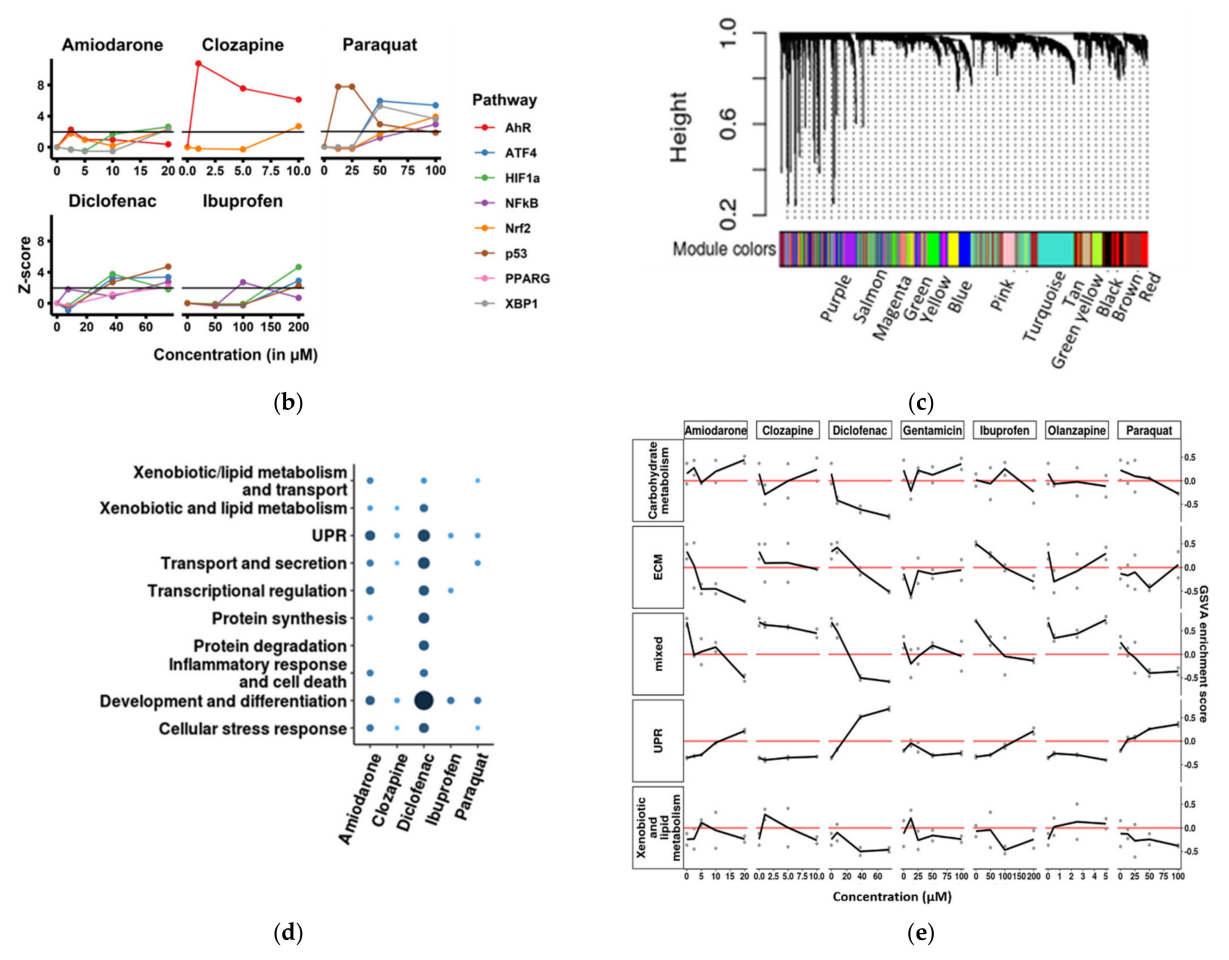
Cellular stress pathway genes are highly differentially expressed in SBAD2-3x-AAGLY HLCs upon chemical treatment. (**a**) Heatmaps of the top differentially expressed genes in response to treatment for each of the chemicals. X-axes represent increasing chemical concentrations. For each gene the CPMs of replicate samples were averaged and transformed into Z-scores for visualisation. (**b**) Z-score profiles of pathways enriched in response to chemical treatments. (**c**) WGCNA analysis on the data revealed different modules of co-expressed genes (modules shown in Figure S8). Height of the dendrogram is a measure of dissimilarity between genes. Hence, genes at the ‘tips’ of the branches are more similar and more cluster-central. Colour scheme represents the partitioning of the genes into the modules. (**d**) Bubble plot of the modules showing relative number of differentially expressed genes per chemical in each module. (**e**) GSVA plot of selected clusters showing concentration-dependent module activity for each drug. Red horizontal lines indicate GSVA scores of 0, which indicates no over- or underrepresentation of a group of genes. Higher GSVA values indicate that the respective module’s genes are generally higher expressed upon treatment with the respective chemical and concentration, and vice versa.

The probeset panel and raw counts matrix obtained from TempO-Seq on treated SBAD2-3x-AAGLY-HLCs is provided in Tables S6 and S7 respectively. We used DESeq2 to fit generalised linear models to model the effect of chemicals at different concentrations on gene expression (Figure 3a). Differential expression analysis revealed large numbers of differentially expressed genes upon treatment with diclofenac (i.e., 552 genes) and amiodarone (i.e., 109 genes). Only low to moderate numbers of genes were differentially expressed after treatment with paraquat (i.e., 15 genes), ibuprofen (i.e., 13 genes), and clozapine (i.e., 7 genes). Numerous genes, known to be involved in hepatotoxicity, were induced upon treatment with amiodarone, diclofenac, and ibuprofen [40]. These included *DDIT3* (UPR), *JMJD6* (p53 pathway), *HSPA6* and *ATF4* (UPR), and *TRIB3* (NFKB associated), and *GDF15* (oxidative stress and acute injury). *INSIG1* (cholesterol biosynthesis), which is known to be involved in the development of metabolic disorders upon treatment with clozapine [51], was differentially expressed in cells treated with clozapine. Additionally, *S100P*, which is an early urinary biomarker of acute kidney toxicity caused by ibuprofen [52], was differentially expressed upon treatment with ibuprofen. Significant differential expression was not observed after 72 h of treatment, which may be due to a washout effect after medium change. The enrichment of stress response pathways in the DEGs was determined using the gene-pathway annotations made for the TempO-Seq probeset for eight common stress response pathways by Wellens et al. [33], and the enrichment z-scores were plotted against the concentration of chemical treatment (Figure 3b). The pathways were named after key transcription factors in each pathway, i.e., ATF4 and XBP1 for different arms of ER stress pathway, HIF1A, PPARG, and NRF2 for oxidative stress responses, AhR pathway for xenobiotic response, and the P53 pathway for cell death mechanisms. Based on the enrichment z-scores, majority of the DEGs in response to the treatments were enriched for oxidative stress and ER stress/UPR pathway responses.

As differential expression analysis tools give merely a ranking of significantly differentially expressed genes, while gene co-expression analysis techniques cluster genes into co-expression modules that are enriched for specific pathways, we also applied WGCNA analysis on our dataset to identify modules that were enriched upon treatment with the different chemicals. WGCNA gene co-expression analysis revealed 13 co-expression modules (Figure 3c). We used enriched GO terms to assess each module’s function. The black and red modules were found to be involved in lipid and xenobiotic metabolism and transport, and the green-yellow module contained genes involved in carbohydrate metabolism. The turquoise cluster contained genes involved in UPR, and the tan and green clusters contained genes involved in extracellular matrix (ECM) and transport and secretion, respectively. The yellow and pink modules contained genes involved in protein synthesis and protein degradation, respectively, while the magenta module contained genes involved in transcription regulation. The blue module had GO terms associated with development and differentiation, and the brown and purple modules with cellular stress response and inflammatory response/cell death, respectively. The salmon module had mixed GO terms. The GO molecular functions of all modules are presented in Figure S8. The relative number of differentially expressed genes in each module is represented in Figure 3d.

Next, we assessed the activity of each module for each chemical with increasing concentrations. Activity was assessed using the GSVA R package (Figure 3e). Upon treatment with diclofenac, amiodarone, or ibuprofen, the UPR module activity increased with increasing concentrations, while expression of genes in the mixed and ECM modules decreased. Increased UPR gene expression was also observed upon treatment with increasing concentrations of paraquat. Activity of the carbohydrate metabolism module as well as the xenobiotic metabolism model was downregulated by diclofenac. Olanzapine and gentamicin treatment had limited effects on the GSVA scores, in line with the fact that they did not cause cytotoxicity in HLCs. 

We also compared the expression in the TempO-Seq data of genes responsible for xenobiotic metabolism and transport in HLCs vs HepaRGs and sandwich-cultured PHHs and plotted the log2CPM values in Figure S9. In general, the SBAD2-3x-AAGLY-HLCs expressed lower levels of these genes in comparison to HepaRGs and PHHs, except for *CYP2D6* and *ABCB11* which were lower in HepaRG, and *CYP1A2*, *PPARA* and *HNF4A* which were not significantly different between SBAD2-3x-AAGLY-HLCs and HepaRG.

### 3.4. Cellular Stress Genes Show Differential Expression at Different Time-Points after Chemical Treatment

**Figure 4:**
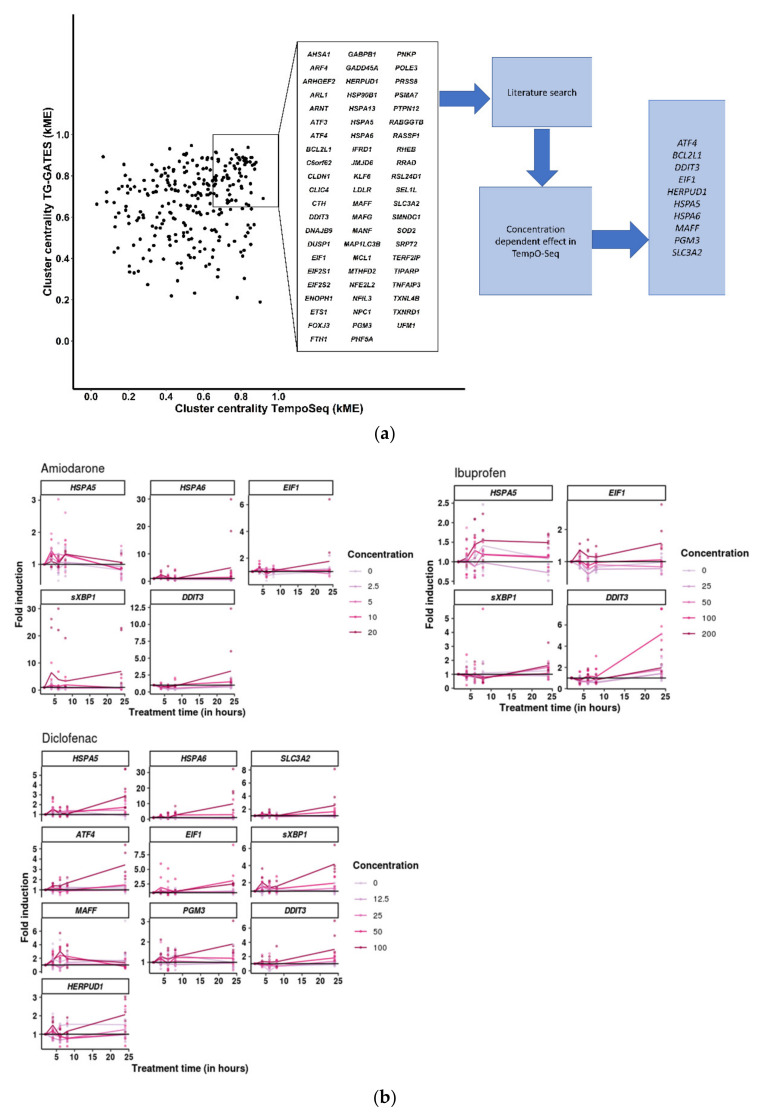
Cellular stress genes show differential expression at different time-points after chemical treatment. (**a**) Cluster centrality plot of genes from the UPR cluster from TempO-Seq for amiodarone, diclofenac, and ibuprofen, vs. genes from selected clusters of TG-GATES. The top cluster central genes (kME > 0.65) were selected and then narrowed down to 10 genes that had higher literature evidence for toxicity. (**b**) Differential expression of selected genes in d40 SBAD2-3x-AAGLY HLCs in response to chemical treatments for each time-point. Coloured lines indicate different concentrations of the respective chemical. X-axes indicate the treatment times.

To assess if cluster-central cellular stress genes from the SBAD2-3x-AAGLY-HLCs-TempO-Seq and TG-GATES-PHH analyses displayed dose- and time-dependent expression, we selected 10 genes based on three criteria (genes listed in Table S8): (1) a cluster-centrality measure (kME) higher than 0.65 in selected clusters (see Section 2) of both the SBAD2-3x-HLC-TempO-Seq and TG-GATES-PHH analyses, (2) a significantly increasing induction of expression with increasing concentrations of at least one chemical, and (3) literature supporting the gene relevance (Figure 4a).

Day 40 SBAD2-3x-AAGLY-HLCs were treated with amiodarone, diclofenac, or ibuprofen at doses mentioned in the Section 2. Cell lysates collected after chemical addition were analysed for gene expression using RT-qPCR. Diclofenac induced significant differential expression of *ATF4*, *DDIT3*, *EIF1*, *HERPUD1*, *HSPA5*, *HSPA6*, *MAFF*, *PGM3*, and *SLC3A2*, at different concentrations and time-points. Amiodarone treatment resulted in significant differential expression of *DDIT3*, *HSPA5*, *EIF1*, and *HSPA6*, while ibuprofen treatment significantly altered the expression of *DDIT3*, *EIF1*, and *HSPA5* (Figure 4b and Figure S10). For the different components of the UPR stress pathway, we observed an early-stage increase in transcripts for the chaperone proteins, *HSPA5* and *HSPA6*, with in some cases also an increased expression later after chemical exposure, possibly the result of gene induction by the downstream effectors [53]. We also observed an early increase in *MAFF* (which is responsible for antioxidant and cytoprotective responses). In general, significant increases in genes functioning downstream in the pathway, such as *EIF1*, *DDIT3* and *sXBP1*, were observed at later time-points of chemical exposure and were induced more highly in response to higher chemical concentrations. All effects were assessed with linear models (significance test results are in Table S10).

Thus, the ER stress/UPR mechanisms were induced at both early and late time-points after amiodarone, diclofenac, or ibuprofen treatment, including an increased expression of *PERK-EIF2A-ATF4* pathway, and induction of *DDIT3* indicating the initiation of apoptotic cascades. In addition, spliced *XBP1* transcripts were also increased at higher concentrations/times by all three chemicals, indicating the activation of the IRE1 cascade.

### 3.5. Benchmarking

**Figure 5:**
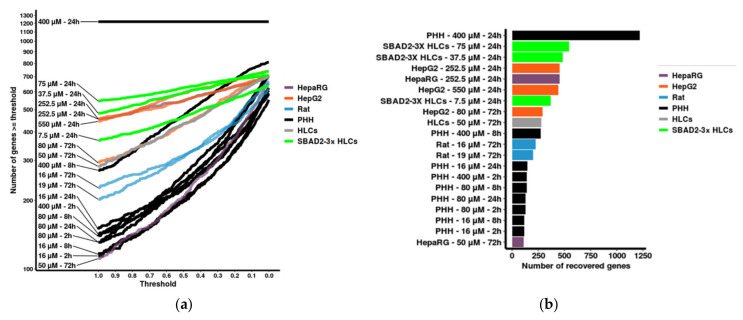
Benchmarking of the SBAD2-3x-AAGLY HLC model versus other hepatocyte models for transcriptional changes induced by diclofenac. (**a**) Recovery curves indicating the number of genes for which the fold induction (upon diclofenac treatment in PHH treated with 400 μM diclofenac and for a duration of 24 h) was recapitulated by each respective model, under various thresholds of a similarity metric calculated as $S_{i}=\frac{log_{2}FC_{model}}{log_{2}FC_{gold}}.$ A gene *i* was counted when $S_{i}\geq Threshold.$ (**b**) Bar chart indicating for each hepatocyte model the number of genes with a fold induction (upon diclofenac treatment) of equal or higher magnitude (i.e., $S_{i}\geq 1$ ) compared to the fold induction in PHH (treated with 400 μM diclofenac and for a duration of 24 h).

Finally, we sought to benchmark the TempO-Seq analysis of the SBAD2-3x-AAGLY-HLCs with transcriptomics data from other hepatocyte toxicity models. We included in this comparison the published transcriptomics data on HepaRG cells, HepG2 cells, hESC-HLCs, primary rat hepatocytes (with and without Trichostatin A added for epigenetic stabilisation) and PHH from the TG-GATES together with the SBAD2-HC3x-HLCs TempO-Seq data. Only dose responses to diclofenac were found in all datasets. Cellular toxicological responses involve specific transcriptomic changes. Hence, it follows that similar transcriptomic changes occur in similar models. To measure this, we calculated the number of genes induced by diclofenac under a range of thresholds on the similarity $S_{i}$ (defined in the Section 2) in published models and the SBAD2-3x-AAGLY-HLCs compared with PHHs (treated with 400 μM for 24 h, i.e., the gold standard). The highest gene recovery (at the highest threshold) was observed for the SBAD2-3x-AAGLY-HLC model treated for 24 h with diclofenac at 75 μM (546/1220 genes (44.75%) and 37.5μM (482/1220 genes (39.51%). This was followed by HepaRG cells treated with 252.5 μM of diclofenac for 24 h, and HepG2 treated with 252.5 μM and 550 μM of Diclofenac for 24 h (Figure 5a,b). Lower gene recoveries were observed for hESC-Heps and primary rat hepatocytes, and the lowest recoveries were observed in the TG-GATES PHHs and HepaRGs treated at lower diclofenac concentrations (i.e., 16–80 μM) or PHHs treated for 2 h.

## 4. Discussion

There is an urgent need to replace animal-based testing models in pre-clinical studies and industrial risk assessment approaches of hepatotoxicity. In vitro models have been optimized to a great degree over the past decades, and continue to be adapted. Freshly isolated primary hepatocytes mimic in vivo hepatocytes the best. However, PHH culture models suffer from inter-individual variation, rapid dedifferentiation and loss of mature hepatocyte characteristics. Liver-derived transformed hepatic cell lines are more stable in culture but display significantly less robust biotransformation than PHHs. Therefore, PSC-derived hepatocytes or HLCs are increasingly being explored for their potential in chemical risk assessment studies.

Here, we used a recently optimized PSC-HLCs model obtained by genome engineering and metabolic engineering of the culture medium [15], to assess acute hepatocellular toxicity. Based on the expression pattern of mature hepatocyte markers at the RNA and protein level, 3x-AAGLY-HLCs appeared more mature than HepG2 cells but not as mature as HepaRGs and PHHs (Figure 1, Figures S7 and S9). Of note, SBAD2-3x-AAGLY-HLCs displayed lower BFC metabolization than HLC progeny from the 3x-AAGLY-hESC line and two other 3x-AAGLY-hiPSC lines we described previously [15,54]. In addition, these previous studies also demonstrated higher CYP3A4 drug biotransformation by 3x-AAGLY-ESC-derived HLCs than HepaRG cells. These differences might be caused by donor-specific differences. Nevertheless, the SBAD2-3x-AAGLY-HLCs, can correctly classify agents causing acute hepatocellular toxicity.

As cytotoxicity dose-response studies are increasingly being replaced by high-throughput studies to gain mechanistic information for improved toxicity prediction, we used TempO-Seq as a tool to perform high-throughput RNA-sequencing studies from low starting material. The transcriptomics data revealed that these chemical treatments induce early-time point responses of cellular stress, including ER stress, UPR, and oxidative stress. In the past decade, several studies identified ER stress and subsequent UPR as one of the major molecular initiating events in response to chemical-induced toxicity in the liver [40,55,56], as when unmitigated or prolonged, UPR stress causes deleterious downstream effects, including lipid accumulation (steatosis), cell death and inflammatory signalling [57]. We selected the highly cluster-central genes from the WGCNA analysis from our dataset and TG-GATES dataset, with evidence in literature for their involvement in cellular stress signalling. These genes were then shown to have time and concentration-dependent differential regulation upon treatment with amiodarone, diclofenac, and ibuprofen.

In the event of ER stress, the unfolded protein response is initiated as an adaptive response [53]. When the chaperone HSPA5 dissociates from the three membrane effectors, activating transcription factor 6 (ATF6), PKR-like ER kinase (PERK), and inositol requiring enzyme 1 (IRE1), these proteins enter an activated state by cleavage (ATF6) or phosphorylation (IRE1 and PERK), and a series of downstream events ensue to restore cellular homeostasis. Phosphorylation of eukaryotic initiation factor 2-alpha (eIF2a) by PERK leads to decreased translation and selective activation of transcription of activating transcription factor 4 (ATF4), which in turn induces the expression of chaperon synthesis and autophagy. Activated IRE1 co-translationally splices out a 26 bp region from *XBP1* mRNA, the protein product of which is a transcription factor. ATF6 promotes the transcription of genes such as chaperons and proteins that are part of ER-associated degradation (ERAD). When these mechanisms prove inadequate in resolving ER stress, apoptosis may result via C/EBP homologous protein (CHOP/DDIT3). We demonstrated the activation of the different axes of the ER stress/UPR cascade, namely the PERK-EIF2A-ATF4 and the IRE1, as well as the apoptosis pathways upon exposure of the SBAD2-3x-AAGLY-HLCs to amiodarone, diclofenac, or ibuprofen for up to 24 h at doses lower than IC10.

Relatively few transcriptomics datasets exist on chemical-treated cells, and the majority of these are microarray datasets. Therefore, when we sought to benchmark the transcriptomics changes in the SBAD2-3x-AAGLY-HLCs model against other models, diclofenac was the only chemical in common in all. We benchmarked the TempO-Seq data in response to diclofenac from the SBAD2-3x-AAGLY-HLCs model versus transcriptomics studies of other models, including HepG2 cells, HepaRG cells, ESC-HLCs, and PHH described by [16,38,47], respectively. This demonstrated that the SBAD2-3x-AAGLY-HLCs identify more differentially expressed genes as compared to transcriptomics data from the other models at lower concentrations of diclofenac treatment. Primary rat hepatocytes appeared to be less sensitive in detecting differentially expressed genes, which may be due to differences in drug biotransformation [58]. The lower sensitivity of PHHs may be due to the rapid de-differentiation of PHHs when cultured even for 12–24 h [59], and/or the well-known variability between different PHH donors in susceptibility to chemical toxicity [8].

Transcriptomics methods in combination with improved cellular models can thus be useful for detection of early cellular changes in response to treatments with toxic chemicals. Ideally, risk assessment decisions should be made using a model consisting of both parenchymal and non-parenchymal cells, which is more representative of the complexity of the liver. In recent years, advances have been made in the optimization of human-relevant iPSC-derived three-dimensional (3D) spheroid [60,61,62] and organoid [63,64,65] models for long-term toxicological studies, with or without NPCs in co-culture. The Verfaillie lab has also recently described a hydrogel-based 3D co-culture system with improved function and maturation [54]. It will be of interest to differentiate 3x-AAGly-HLCs in combination with iPSC-derived non-parenchymal cells in 3D, for longer term and repeat-dose toxicity studies, and to test its applicability for assessment of toxicity risk and mechanisms. However, transcriptomics and other omics readouts of these complex models, unless done at the single cell level, are much less straightforward to interpret, and cannot pinpoint which cell is responsible for the stress response. Hence, such models should ideally also be equipped with genetically encoded fluorescent reporter genes to enable determination of the contribution of parenchymal and non-parenchymal cells to the toxicity response. Such models, however, are currently not yet available, even if this might become possible in the coming years. On a final note, the information obtained from transcriptomics methods are limited to adaptive stress responses in the form of differential expression of specific transcripts. Hence, a combinatorial approach with different omics techniques may offer more insights into the connections between the molecular events and the outcome of cell death. For instance, transcriptomics, proteomics, and metabolomics have been used in combination recently for understanding toxicity mechanisms of perfluorohexanoic acid [66] and dictamnine [67] in mice. Such combined approaches could also further enhance risk assessment decisions when applied to PSC-HLC models in future studies.

## Data Availability

Not applicable.

## Supplementary Materials

The following are available online at https://www.mdpi.com/article/10.3390/toxics10010001/s1, Supplementary Methods; Figure S1: Genome engineering for generation of the SBAD-3x iPSC line, Figure S2: Characterization of the SBAD2-FRT line for pluripotency. Figure S3: Profiling for genomic aberrations and SNPs. Figure S4: Confirmation of correct insertion of the FRT cassette in the SBAD2-FRT line. Figure S5: Confirmation of insertion of 3x cassette in the SBAD2-3x lines. Figure S6: Protocol for differentiation to HLCs. Figure S7: Immunofluorescent staining of HepG2 cells. Figure S8a,b: GO Molecular Functions for clusters obtained from WGCNA (Figure 3b), Figure S9: Expression levels of genes in SBAD2-3x-HLCs involved in phase I and phase II metabolism and mature hepatocyte markers from TempO-Seq data, relative to PHHs and HepaRGs. Figure S10: Cellular stress genes show differential expression at different time-points after treatment of SBAD2-3x-AAGLY HLCs with Amiodarone. Table S1: PCR primers and southern blot probes. Table S2: media compositions. Table S3: Primer sequences used for RT-qPCR. Table S4: Antibodies used for immunofluorescent staining. Table S5: Chemicals chosen for dose-response assay treatments and drug-induced liver injury (DILI) likelihood scores. Table S6: TempO-Seq treatment concentrations. Table S7: TempO-Seq probes and corresponding genes. Table S8: raw counts matrix obtained from TempO-Seq on the HLCs treated with chemicals for different concentrations and time-points. Table S9: Cluster-central genes chosen based on differential expression in TempO-Seq data and evidence and relevance in chemical-induced toxicity. Table S10: linear modelling of concentration and time effects on gene expression.
