# Supplementary material for: HiPSC-Derived Hepatocyte-like Cells Can Be Used as a Model for Transcriptomics-Based Study of Chemical Toxicity
Source: Toxics. 2021 Dec 21;10(1):1. doi: 10.3390/toxics10010001 (PMC8780865; doi:10.3390/toxics10010001)
Supplement: Supplementary file 1 [file toxics-10-00001-s001.zip › toxics-1429150 sp 1.12/toxics-1429150 SP 1.12.pdf]

toxics

MDPI

Supplementary Materials: HiPSC-Derived Hepatocyte-Like Cells Can be Used as a Model for Transcriptomics-Based Study of Chemical Toxicity

Sreya Ghosh, Jonathan De Smedt, Tine Tricot, Susana Proença, Manoj Kumar, Fatemeharefeh Nami, Thomas Vanwelden, Niels Vidal, Paul Jennings, Nynke I. Kramer and Catherine M. Verfaillie

Supplementary methods

1. Characterisation of the SBAD2-FRT and SBAD2-3X iPSC Lines (Figures. S2-S5):

1.2 Quantitative Analysis of Trilineage Differentiation:

SBAD2-FRT iPSCs were differentiated to embryoid bodies to test for their ability to differentiate to all three lineages. Differentiation to the three germ layers and loss of pluripotency was analysed using the ScoreCard methodology (Thermo Fisher Scientific) according to manufacturer instructions [1].

Immunofluorescence staining for pluripotency markers: Fixed undifferentiated SBAD2-FRT iPSCs were stained for pluripotency markers as described in the Immunofluorescence staining section in Methods, and imaged on an Axioimager Z1 microscope. The antibodies are listed in table S3.

1.3 SNP Profiling:

To demonstrate SBAD2 iPSC identity, a reference panel of 32 SNPs was analysed using TaqMan assay (Life Technologies) on DNA from the SBAD2 and SBAD2-FRT iPSCs. The analysis was done using Genotyper Software.

1.4 Genome Integrity Verification:

The CytoSure Syndrome Plus 60K array (Oxford Gene Technology, Oxford, UK) was used for assessing the genome integrity of the original SBAD2 iPSC line and the SBAD2-FRT iPSC line. Arrays were scanned using an Agilent microarray scanner at 2 µm resolution, followed by calculation of signal intensities using Feature Extraction software (Agilent Technologies). Visualization of results and data analysis was performed using the CytoSure Interpret Software (Oxford Gene Technology) and the circular binary segmentation algorithm.

1.5 Junction PCR:

3′ and 5′ junctions of the *AAVS1* locus were amplified using PCR and visualised on a 1% agarose gel. The primer sets used for the 3′and 5′ junction assay PCR of the SBAD2-FRT iPSCs and the 3′junction assay for the SBAD2-3x line are listed in table S1.

1.6 Southern Blot:

To determine whether the genomic integration of the FRT cassette was mono or biallelic, southern blotting was performed on the SBAD2-FRT iPSC. Briefly, the 5′ internal probe (table S1) was labelled by PCR using Digoxigenin-dNTPs (Roche). The genomic DNA was digested using the Nco1 restriction enzyme (NEB) and run on 0.7% agarose gel. The bands were transferred using a capillary flow setup onto a Zeta probe membrane (Biorad). The membrane was hybridised with the probe and exposed to an X-ray film to develop the image.

1.7 Flow Cytometry:

Cells were detached, resuspended in 100 µl buffer (1% bovine serum albumin in PBS) and analyzed on a Canto HTS flow cytometer (BD Biosciences) to determine the respective fractions of GFP positive or negative cells. After exclusion of cell doublets, GFP fluorescence was determined on the FL1-FITC channel. For the SBAD2-FRT iPSCs, the population of cells that expressed GFP was quantified, as a sign of presence of the inserted cassette. Following RMCE of the transcription factor cassette, the SBAD2-3x iPSCs were again analysed by FACS to demonstrate removal of the GFP cassette (percentage of GFP-negative cells). Data was analysed using FlowJo software (BD Biosciences).

Table S1. PCR primers and southern blot probes.

Primer/Probe	Sequence	Primer Direction	PCR Cycle
5'JA-FRT line	CACTTTGAGCTCTACTGGCTTC	forward	95 °C, 5′–[95 °C, 30′′–68 °C (−0.5 °C/cycle), 1′ 30′′]X15–[95 °C, 30′′–58 °C, 30′′–72 °C, 1′ 30′′]X25–72 °C, 5′
	CGTTACTATGGGAACATACGTCA	reverse	
3'JA-FRT line	TAACTGAAACACGGAAGGAG	forward	95 °C, 5′–[95 °C, 30′′–68 °C (−0.5 °C/cycle), 1′ 30′′]X15–[95 °C, 30′′–58 °C, 30′′–72 °C, 1′ 30′′]X25–72 °C, 5′
	AAGGCAGCCTGGTAGACA	reverse	
5' JA-3X line	CACTTTGAGCTCTACTGGCTTC	forward	95 °C, 5′–[95 °C, 30′′–72 °C (−0.5 °C/cycle), 1′ 30′′]X15–[95 °C, 30′′–64 °C, 30′′–72 °C, 1′ 30′′]X25–72 °C, 5′
	CATGTTAGAAGACTTCCTCTGC	reverse	
5′ Internal probe (southern blot)	CTTTCTCTGACCTGCATTCTC	forward	**Labelling Cycle**
	CTGCCCAAATGAAAGGAGT	reverse	95 °C, 2′–[95 °C, 30′′ –58 °C, 30′′–72 °C, 45′′]X30–72 °C, 7′

Table S2. Media compositions.

(a) Liver differentiation medium (LDM).

Liver Differentiation Media (LDM)	Catalog Number	Supplier	Volume for 500 mL (mL)
DMEM LG	31885	Invitrogen	285
MCDB pH=7.2	M6770	Sigma	200
Pen/Strep	15140	Invitrogen	5
L-Ascorbic Acid	A8960	Sigma	5
ITS	4140–045	Invitrogen	1.25
LA-BSA	L9530	Sigma	1.25
β-Mercapto	31350	Invitrogen	0.5
Dexamethasone	D-2915	Sigma	2

(b) LDM with amino acids (LDMAA).

Liver Differentiation Media with Amino Acids (LDMAA)	Catalog Number	Supplier	Volume for 650 mL (mL)
LDM			500
MEM Amino Acids Solution (50X)	11130051	Gibco	50
MEM Non-Essential Amino Acids Solution (100X)	11140035	Gibco	100

(c) LDM with amino acids and additional glycine (LDMAAGLY).

Liver Differentiation Media with Amino Acids Including Glycine (LDMAAGLY)	Catalog Number	Supplier	Volume for 650 mL (mL)
LDM			500
MEM Amino Acids Solution (50X)	11130051	Gibco	50
MEM Non-Essential Amino Acids Solution (100X)	11140035	Gibco	100
Glycine	G8898	Sigma	2 g

Table S3. Primer sequences used for RT-qPCR.

Primer	Sequences
RPL19-forward	ATTGGTCTCATTGGGGTCTAAC
RPL19-reverse	AGTATGCTCAGGCTTCAGAAGA
ALB-forward	TGGCACAATGAAGTGGGTAA
ALB-reverse	CTGAGCAAAGGCAATCAACA
HNF1A-forward	ACACCTCAACAAGGGCACTC
HNF1A-reverse	TGGTAGCTCATCACCTGTGG
CYP3A4-forward	TTCCTCCCTGAAAGATTCAGC
CYP3A4-reverse	GTTGAAGAAGTCCTCCTAAGCT
CYP2C9-forward	TGGACATGAACAACCCTCA
CYP2C9-reverse	ACTGCAGTGTTTTCCAAGC
HNF4A-forward	ACTACGGTGCCTCGAGCTGT
HNF4A-reverse	GGCACTGGTTCCTCTTGTCT
AFP-forward	TGAGCACTGTTGCAGAGGAG
AFP-reverse	GTGGTCAGTTTGCAGCATTC
G6PC-forward	GTGTCCGTGATCGCAGACC
G6PC-reverse	GACGAGGTTGAGCCAGTCTC
HNF4A-forward	ACTACGGTGCCTCGAGCTGT
HNF4A-reverse	GGCACTGGTTCCTCTTGTCT
NTCP-forward	AACCTCAGCATTGTGATGAC
NTCP-reverse	GTATTGTGGCCGTTTGGA
PEPCK-forward	AAGAAGTGCTTTGCTCTCAG
PEPCK-reverse	CCTTAAATGACCTTGTGCGT
ATF4-forward	ATGACCGAAATGAGCTTCCTG
ATF4-reverse	GCTGGAGAACCCATGAGGT
BCL2L1-forward	GAGCTGGTGGTTGACTTTCTC
BCL2L1-reverse	TCCATCTCCGATTCAGTCCCT
DDIT3-forward	GGAAACAGAGTGGTCATTCCC
DDIT3-reverse	CTGCTTGAGCCGTTCATTCTC
EIF1-forward	GAAACGGCAGGAAGACCCTTA
EIF1-reverse	CGGATGCTCAATTACAGTACCAT
HERPUD1-forward	ATGGAGTCCGAGACCGAAC
HERPUD1-reverse	TTGGTGATCCAACAACAGCTT
HSPA5-forward	CATCACGCCGTCCTATGTCG
HSPA5-reverse	CGTCAAAGACCGTGTTCTCG
HSPA6-forward	GATGTGTCGGTTCTCTCCATTG
HSPA6-reverse	CTTCCATGAAGTGGTTCACGA
MAFF-forward	TCACCGCATGACTGGGTTTT
MAFF-reverse	CTGAGTTTTGTTGTGGGGCG
SLC3A2-forward	TGAATGAGTTAGAGCCCGAGA
SLC3A2-reverse	GTCTTCCGCCACCTTGATCTT
sXBP1_forward	TGCTGAGTCCGCAGCAGGTG
sXBP1_reverse	GCTGGCAGGCTCTGGGGAAG

Table S4. Antibodies used for immunofluorescent staining.

Antibody	Company	Catalog Number	Dilution
Mouse anti-HNF4α (K9218)	Abcam	ab41898	1:200
Anti-Human CYP3A4	Tebu-Bio (Cypex)	PAP 011 (150616)	1:100
Mouse anti-AFP	R&D Systems	MAB 1368	
Rabbit IgG	BD Pharmingen	550875 (6175659	Isotype
Donkey anti-Mouse IgG (H+L) Highly Cross-Adsorbed Secondary Antibody Alexa Fluor 488	Molecular probes	A-11029	1/500
Donkey anti-Rabbit IgG (H+L) Highly Cross-Adsorbed Secondary Antibody. Alexa Fluor 555	Molecular probes	A21429	1/500
Rabbit anti-Oct4 (H-134)	Santa Cruz	SC-9081	1/100
Rabbit anti-SOX2	Chemicon	AB5603	1/500
Goat-anti Nanog	R&D Systems	AF1997	1/400
Mouse-anti TRA-1-60	Millipore	MAB4360	1/1000
Mouse anti-TRA-1-81	Cell signaling	4745	
Mouse anti-SSEA-1	Santa Cruz	sc-21702	1/200

Table S5. Chemicals chosen for dose-response assay treatments and drug-induced liver injury (DILI) likelihood scores

a: Dose-response assay treatments.

Chemical	CAS Number	Supplier and Catalogue Number	Vehicle	Cmax or Dosing Reference	Cmax, if Applicable (μM)	Treatment Concentrations
Amiodarone hydrochloride	19774-82-4	Sigma A8423	DMSO	[2]	0.806	3.21 μM–200 μM
Busulfan	55-98-1	Sigma B2635	DMSO	[2]	0.276	16.6 μM–500 μM
Clozapine	5786-21-0	Sigma C6305	DMSO	[2]	0.948	1.56 μM–50 μM
cyclosporin A	59865-13-3	Sigma 30024	DMSO	[2]	0.773	0.625 μM–40 μM
Diclofenac sodium salt	15307-79-6	Sigma D6899	DMSO	[2]	7.44	14.7 μM–470 μM
Pamidronate disodium hydrate salt	57248-88-1	J&K 295197	water	[2]	7.16	3.21 μM–200 μM
Cerium dioxide	1306-38-3	Sigma 544841	water	[3]		1.6 μg/mL–50 μg/mL
Paraquat dichloride	1910-42-5	Supelco 36541	water	[4]		0.9μM–10 mM
Ibuprofen sodium salt	31121-93-4	Sigma I1892	ethanol	[5]	164	31.25 μM–1 mM
olanzapine	132539-06-1	Sigma O1141	DMSO			3.125 μM–100 μM
doxorubicin hydrochloride	25316-40-9	J&K 113424	water	[6]	~1	0.31μM–20 μM

lead[II]chloride	7758-95-4	Sigma 7758-95-4	water	[7]		1.6 μM–100 μM
Gentamicin sulfate	1405-41-0	Sigma G1914	water	[8]		60 μM–5 mM
Valproic acid sodium salt	1069-66-5	Sigma P4543	water	[9]	152.4	0.6 mM–10 mM

b: Drug-induced liver injury (DILI) likelihood scores and injury patterns in chosen chemicals.

**Chemical**	**DILI Likelihood Score and Injury Pattern**	**IC50 (μM) in Hepatocellular Models and Reference**	**Toxicity in Other Organs**
Amiodarone	A; hepatocellular injury	45; [10]	Lungs, thyroid
Diclofenac	A; hepatocellular injury	>500; [10]	Kidney
Clozapine	B; hepatocellular and mixed	4.1; [10]	Heart, pancreas
Gentamicin	E	-	Kidney
Olanzapine	No or very rare clinically apparent DILI	-	
Ibuprofen	A; very rare DILI	-	Kidney
Paraquat	Acute liver toxicity		Respiratory tract, kidney
Valproic acid	A; hepatocellular	>100; [10]	Lung, brain
Pamidronate	E	-	Kidney
Cyclosporine	C; mild transient elevations of bilirubin and alkaline phosphate–cholestatic injury	>100; [10]	Kidney, heart
Busulphan	A; sinusoidal obstruction syndrome (dose dependent and prolonged) and cholestatic injury	-	Lung
Doxorubicin	B; transient elevations of enzymes	-	Heart, kidney
Cerium dioxide	No DILI	-	Respiratory tract
Lead Chloride	No DILI	-	Nervous, cardiovascular system, kidney

Likelihood codes: A: well known cause of clinically apparent liver injury; B: likely cause of clinically apparent liver injury; C: probable rare cause of clinically apparent liver injury; D: very rare cases of liver injury; E: unlikely cause of liver injury. -,unavailable

Table S6. TempO-Seq treatment concentrations.

**Chemical**	**Treatment (μM)**	**Time (hours)**
Ibuprofen sodium salt	50, 100, 200	24, 72
Diclofenac sodium salt	7.5, 37.5, 75	24, 72
Clozapine	1, 5, 10	24, 72
Olanzapine	0.5, 2.5, 5	24, 72
Paraquat dichloride	12.5, 25, 50, 100	24
Amiodarone hydrochloride	2.5, 5, 10, 20	24
Gentamicin sulfate	12.5, 25, 50, 100	24

Table S9. Cluster-central genes chosen based on differential expression in TempO-Seq data and evidence and relevance in chemical-induced toxicity.

**Gene**	**Module TempO-**	**Module TG-**	**Fold Change per M Increase in Concentration (Adjusted *p*-value from DESeq2)**	**Function in Toxicity**	**Reference**

	Seq and kME	GATES and kME	Diclofenac	Amiodarone	Ibuprofen		
ATF4	UPR, 0.872	RNA metabolism, 0.832	1.017 (0.0000)		1.003 (0.0001)	induced under ER stress, regulates UPR, oxidative stress response	[11]
BCL2L1	UPR, 0.826	Metabolism (general/ xenobiotic), 0.884	1.011 (0.0002)			Inhibitor/promoter of apoptosis	[12]
DDIT3	UPR, 0.825	UPR/ER stress, 0.796	1.038 (<0.001)	1.029 (0.0432)	1.004 (0.0049)	DNA damage response, UPR response	[11]
EIF1	UPR, 0.824	RNA metabolism, 0.866	1.013 (<0.001)			phosphorylated by PERK, halts protein synthesis	[11]
HERPUD1	UPR, 0.857	UPR/ER stress, 0.889	1.024 (<0.001)			ERAD	[13]
HSPA5	UPR, 0.848	UPR/ER stress, 0.868	1.027 (<0.001)	1.022 (0.0279)		chaperon, master regulator of ER stress pathways	[11]
HSPA6	UPR, 0.774	RNA metabolism, 0.903	1.052 (<0.001)			chaperon	[11]
MAFF	UPR, 0.853	RNA metabolism, 0.889	1.042 (<0.001)			NrF2 pathway, cytoprotective function	[14]
SLC3A2	UPR, 0.850	RNA metabolism, 0.762	1.016 (<0.001)			involved in glutathione synthesis by forming heterotrimer with SLC7A11. Regulator of UPR and apoptosis	[15]
PGM3	UPR, 0.658	UPR and ER stress, 0.793	1.011 (<0.001)			Decrease in phosphoglucomutase activity is correlated with hepatic injury.	[16]

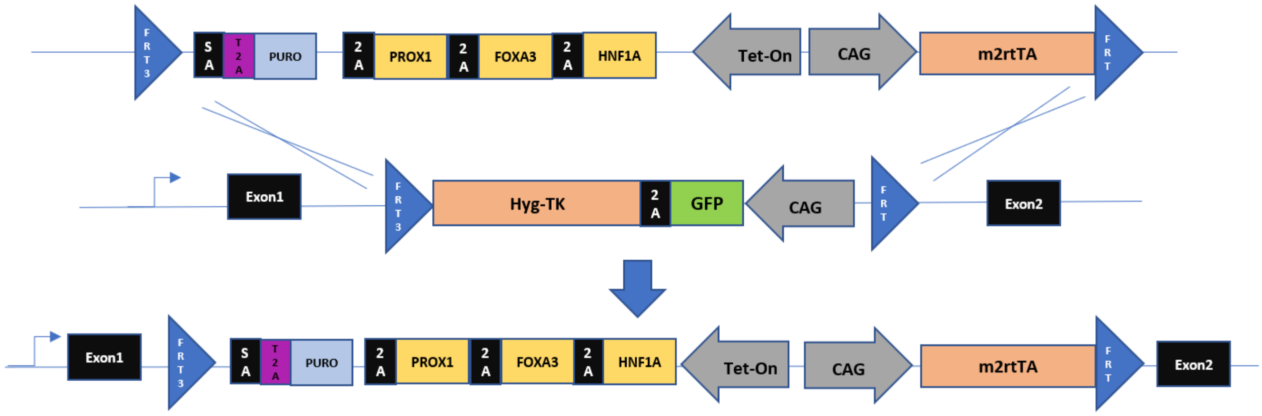

Figure S1. Genome engineering for generation of the SBAD-3x iPSC line. Schematic representation of genome engineering for generation of the SBAD2-3x line. The iPSC line SBAD2-3x was generated by the insertion of Hepatocyte nuclear factor 1-alpha (*HNF1a*), Prospero Homeobox 1 (*PROX1*), and Forkhead box protein A3 (*FOXA3*), using recombinase mediated cassette exchange (RMCE). The upper figure shows the engineered *AAVS1* locus as described in [17], and the bottom figure shows the *AAVS1* with the integrated cassette of the three transcription factors. Briefly, flippase recombines the FRT-flanked cassette with the three transcription factors into the *AAVS1* safe harbor locus using the FRT sites in the SBAD2-FRT line. FRT: Flippase recognition target. Hyg-TK: Hygromycin resistant cassette fused to Thymidine kinase. CAG: chimeric promoter with cytomegalovirus (CMV) enhancer, chicken beta-Actin promoter and rabbit beta-Globin splice acceptor site. Ex: exon. SA: splice acceptor. Puro: puromycin resistance cassette. Tet-On: promoter containing Tetracycline response element (TRE) and CMV minimal promoter. m2rtTA: reverse tetracycline transactivator. LDM: Liver differentiation medium. LDMAA: LDM with amino acids, no glycine added. LDMAAGLY: LDM with amino acids, including glycine. Dox: Doxycycline. BMP4: Bone morphogenetic protein-4. FGF1: Fibroblast growth factor-1. HGF: Hepatocyte growth factor.

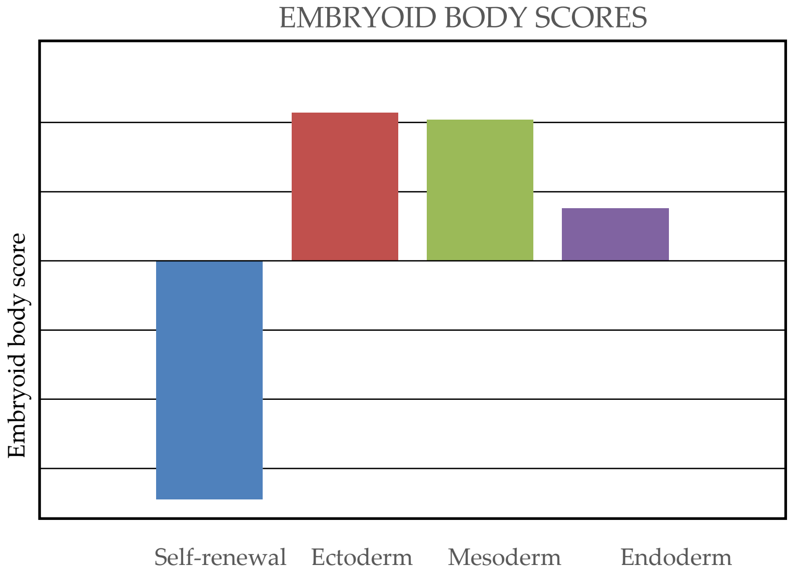

a: SBAD2-FRT clone

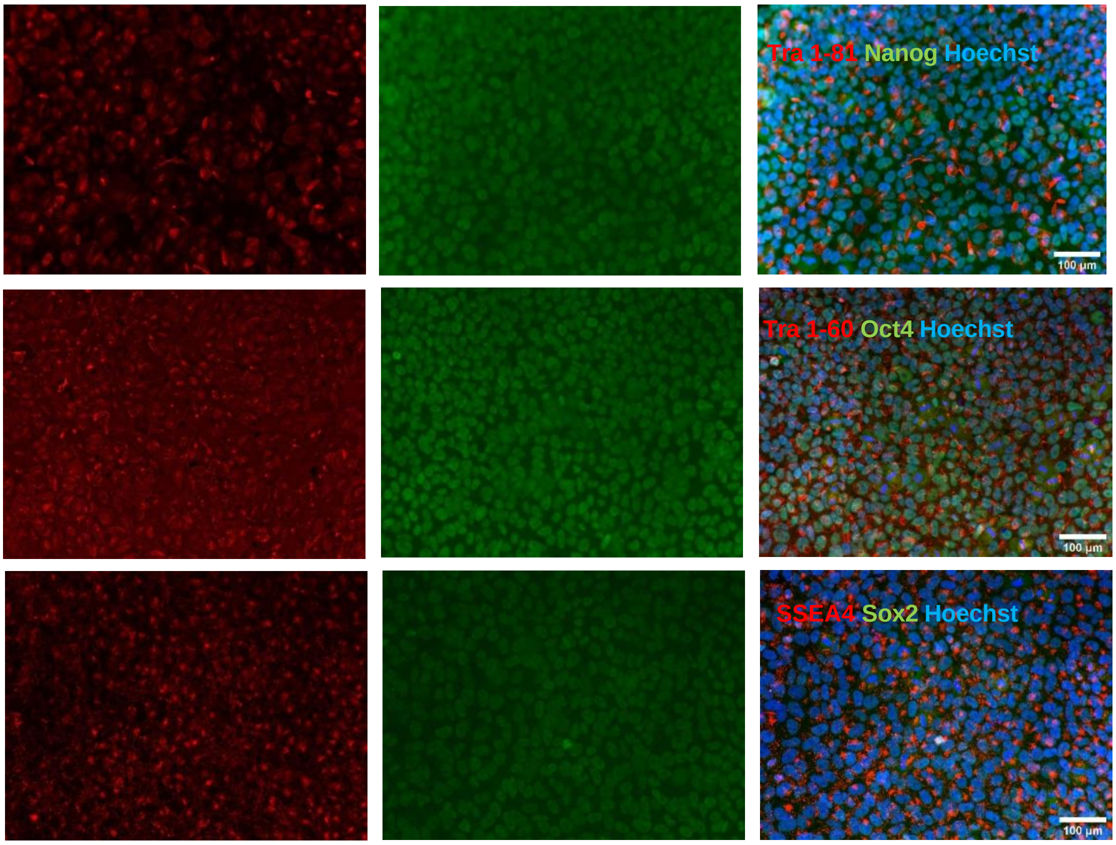

b

Figure S2. Characterization of the SBAD2-FRT line for pluripotency. (**a**). SBAD2-FRT iPSC were differentiated as embryoid bodies for 7 days and then subjected to Scorecard® assay to assess three germ line differentiation. (**b**). Undifferentiated SBAD2-FRT iPSCS were subjected to immunostaining for the pluripotency markers TRA-1-81, NANOG, TRA-1-60, OCT4, SSEA4, and SOX2. Scale bar indicates 100 µm. TRA-1-81: Podocalyxin-like protein, OCT4: POU class 5 homeobox 1, NANOG: nanog homeobox, SOX2: SRY-box transcription factor 2

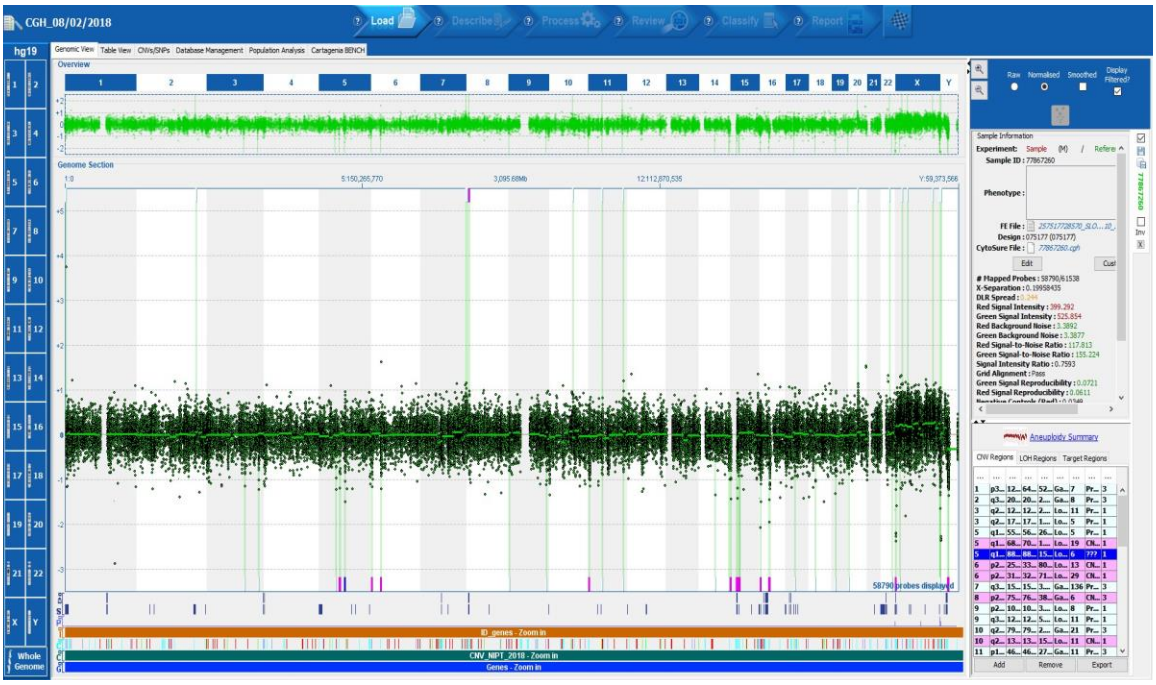

a

	Exp 21	Exp 22	Exp 25
	allele1/allele2	allele1/allele2	allele1/allele2
C___1563023_10	VIC/FAM	VIC/FAM	VIC/FAM
C___1801627_20	VIC/VIC	VIC/VIC	VIC/VIC
C___2728408_10	FAM/FAM	FAM/FAM	FAM/FAM
C___1250735_20	FAM/FAM	FAM/FAM	FAM/FAM
C__15935210_10	VIC/VIC	VIC/VIC	VIC/VIC
C___7431888_10	VIC/FAM	VIC/FAM	VIC/FAM
C___3227711_10	FAM/FAM	FAM/FAM	FAM/FAM
C___1902433_10	VIC/FAM	VIC/FAM	VIC/FAM
C__30044763_10	VIC/FAM	VIC/FAM	VIC/FAM
C__31386842_10	VIC/FAM	VIC/FAM	VIC/FAM
C__33211212_10	VIC/FAM	VIC/FAM	VIC/FAM
C__26524789_10	FAM/FAM	FAM/FAM	FAM/FAM
C__11821218_10	FAM/FAM	FAM/FAM	FAM/FAM
C_____43852_10	VIC/FAM	VIC/FAM	VIC/FAM
C___1670459_10	FAM/FAM	FAM/FAM	FAM/FAM
C___8924366_10	VIC/FAM	VIC/FAM	VIC/FAM
C___1007630_10	VIC/FAM	VIC/FAM	VIC/FAM
C__11522992_10	VIC/FAM	VIC/FAM	VIC/FAM
C___7421900_10	VIC/VIC	VIC/VIC	VIC/VIC
C__10076371_10	VIC/FAM	VIC/FAM	VIC/FAM
C__26546714_10	VIC/FAM	VIC/FAM	VIC/FAM
C___1122315_10	VIC/VIC	VIC/VIC	VIC/VIC
C__27402849_10	VIC/VIC	VIC/VIC	VIC/VIC
C___7457509_10	VIC/FAM	VIC/FAM	VIC/FAM
C__29619553_10	VIC/FAM	VIC/FAM	VIC/FAM
C__11710129_10	VIC/VIC	VIC/VIC	VIC/VIC
C___2953330_10	FAM/FAM	FAM/FAM	FAM/FAM
C___1027548_20	VIC/FAM	VIC/FAM	VIC/FAM
C___8850710_10	VIC/VIC	VIC/VIC	VIC/VIC
C___1083232_10	VIC/VIC	VIC/VIC	VIC/VIC
C__16205730_10	VIC/FAM	VIC/FAM	NOAMP
C___8938211_20	FAM/FAM	FAM/FAM	FAM/FAM

Figure S3. Profiling for genomic aberrations and SNPs. **a**. aCGH of the SBAD2-FRT line detected no genomic aberrations >500 kb. **b**. SNP profiling of the SBAD2-FRT line for a standard SNP panel. SNP: short nucleotide polymorphism.

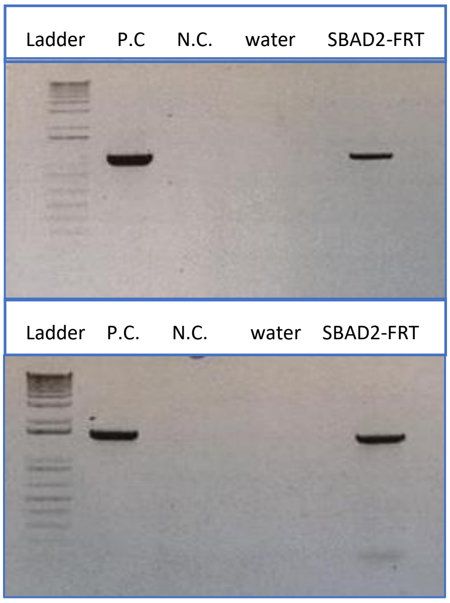

a

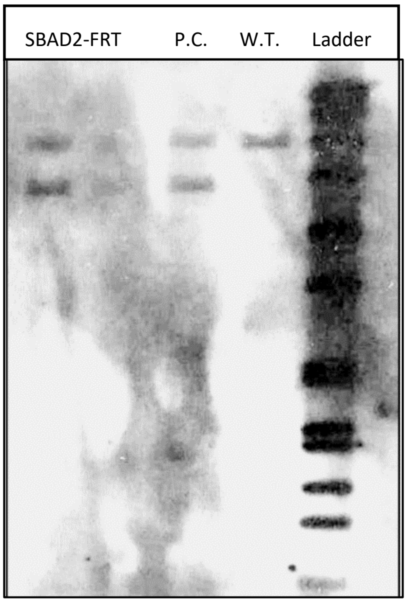

b

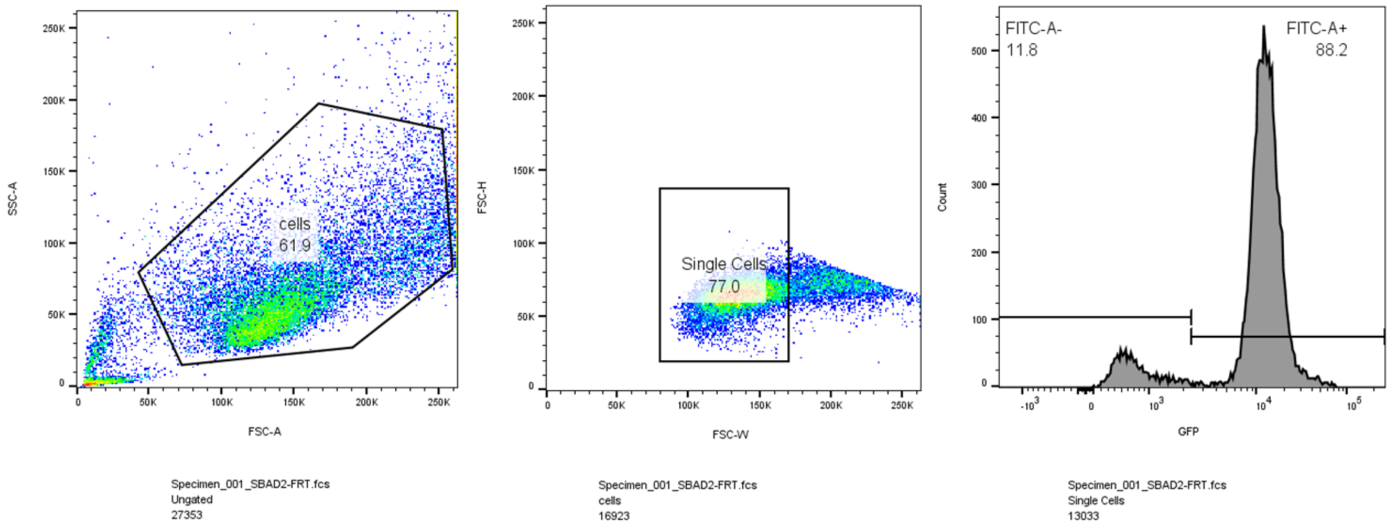

c

Figure S4. Confirmation of correct insertion of the FRT cassette in the SBAD2-FRT line. (**a**). 5′ and 3′ junction PCR assays show correct insertion of FRT cassette in the SBAD2-FRT line when compared to a positive control, i.e. H9 embryonic stem cell line with FRT cassette. Primers used are listed in Table S10. (**b**). Southern blotting using a 5′internal digoxigenin-labelled probe shows mono-allelic insertion of the cassette in the line, and absence of random integrations. Probe sequence listed in Table S10. (**c**). Flow cytometry of the SBAD2-FRT parent line demonstrates presence of 88.2% GFP-positive cells, demonstrating efficient incorporation of the FRT flanked cassette (as described in Figure S1). GFP: green fluorescent protein.

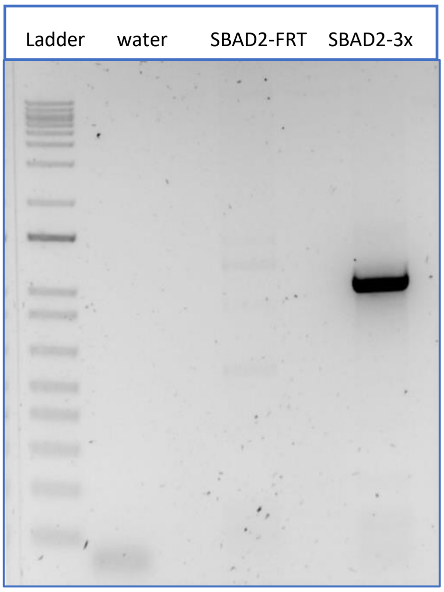

a

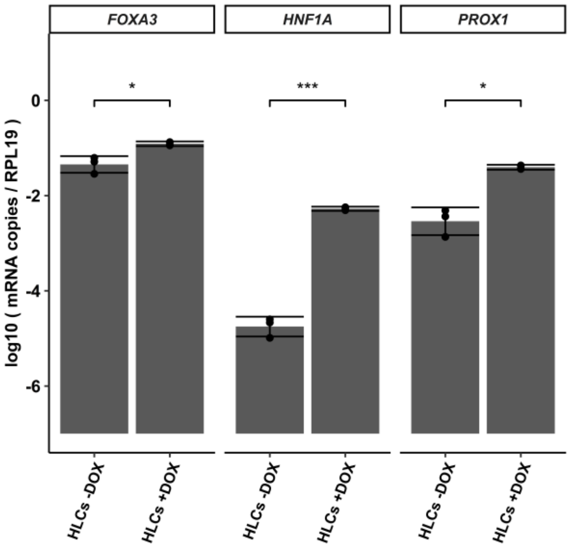

c

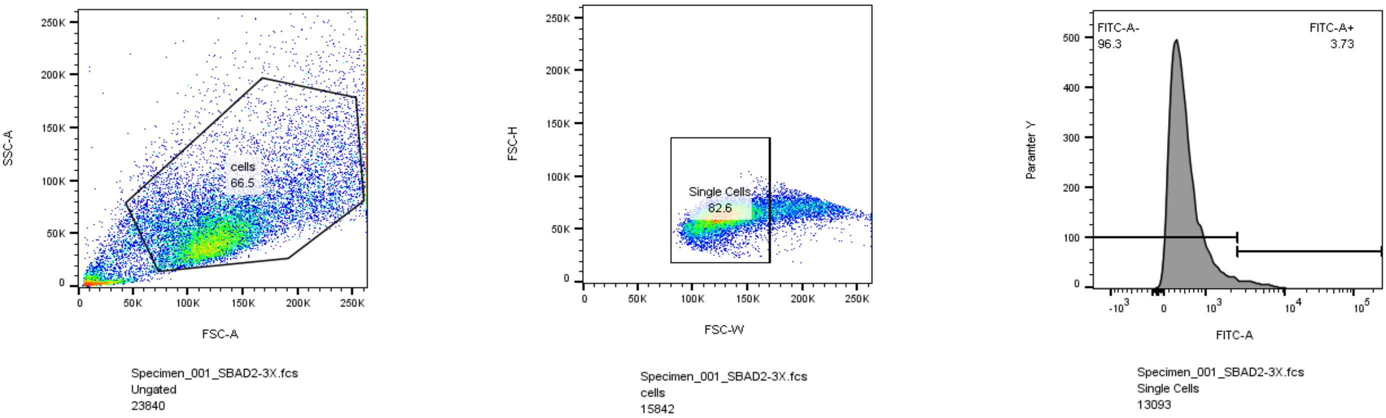

Sample	Percentage GFP + population
SBAD2-3x	3.73 %

b

Figure S5. Confirmation of insertion of 3x cassette in the SBAD2-3x lines. **a**. 5′ junction assay on SBAD2-3x line with primer pairs within the 3x cassette and in the genome demonstrates insertion of the 3x gene cassette in the AAVS1 locus, when compared to negative control (SBAD2-FRT) (primers used are listed in Table S10). **b**. Flow cytometry of the SBAD2-3x line demonstrates that 96.3% of the cells were GFP-negative, demonstrating successful recombination with the inducible TF cassette, (as described in Figure S1). **c**. RT-qPCR for the 3x transcription factors in SBAD2-3x IPSCs differentiated until d20 using AAGLY medium with and without addition of doxycycline shows increased expression upon doxycycline induction of the 3x genes. $n = 2$ independent differentiations. Significance is calculated as compared to SBAD2-3x HLCs without doxycycline by unpaired two-tailed Student's t-test, $^{*}p < 0.05$, $^{**}p < 0.01$.

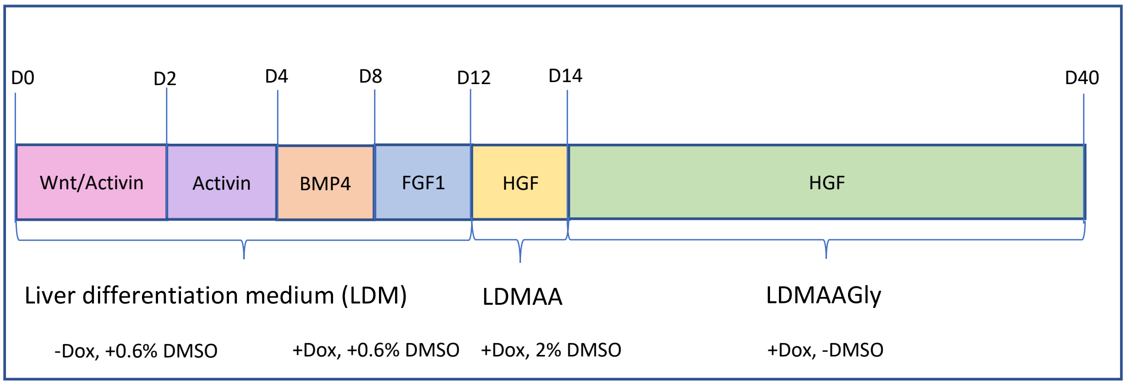

Figure S6. Protocol for differentiation SBAD-3x iPSC to HLCs. Schematic representation of the protocol for the differentiation of SBAD-3x iPSC to hepatocyte-like cells (HLCs). LDM: Liver differentiation medium. LDMAA: LDM with amino acids, no glycine added. LDMAAGLY: LDM with amino acids, including glycine. Dox: Doxycycline. BMP4: Bone morphogenetic protein-4. FGF1: Fibroblast growth factor-1. HGF: Hepatocyte growth factor.

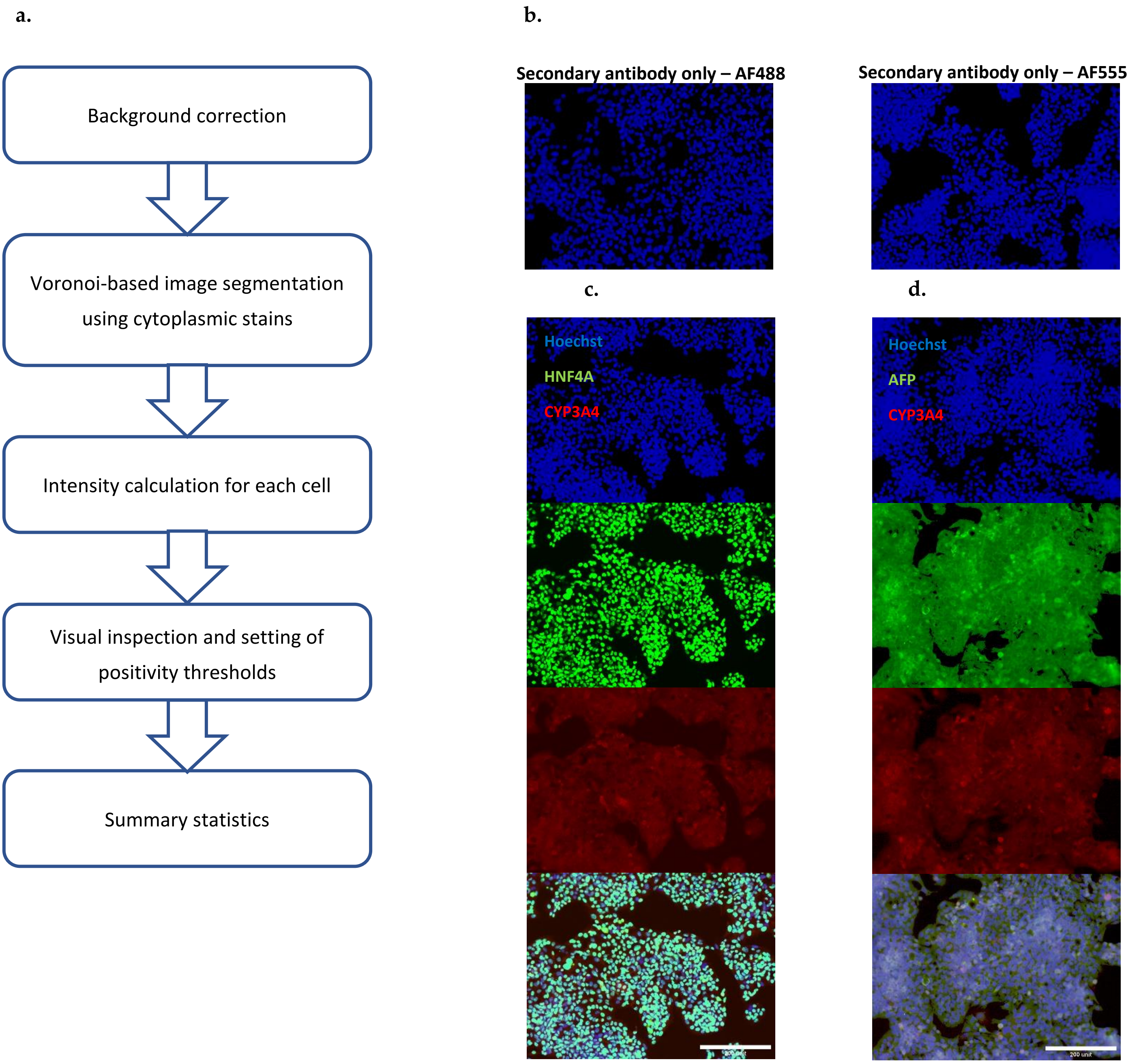

Figure S7. Immunofluorescent staining of HepG2 cells. **a**. Schematic workflow of quantification of imaging done using EBImage and dplyer R packages. **b**. Secondary antibody-only controls for Alexa fluor 488 and Alexa fluor 555. **c**. HepG2 cells were stained for CYP3A4 and HNF4A **d**. HepG2 cells were stained for CYP3A4 and AFP. Scale bar: 200 µm.

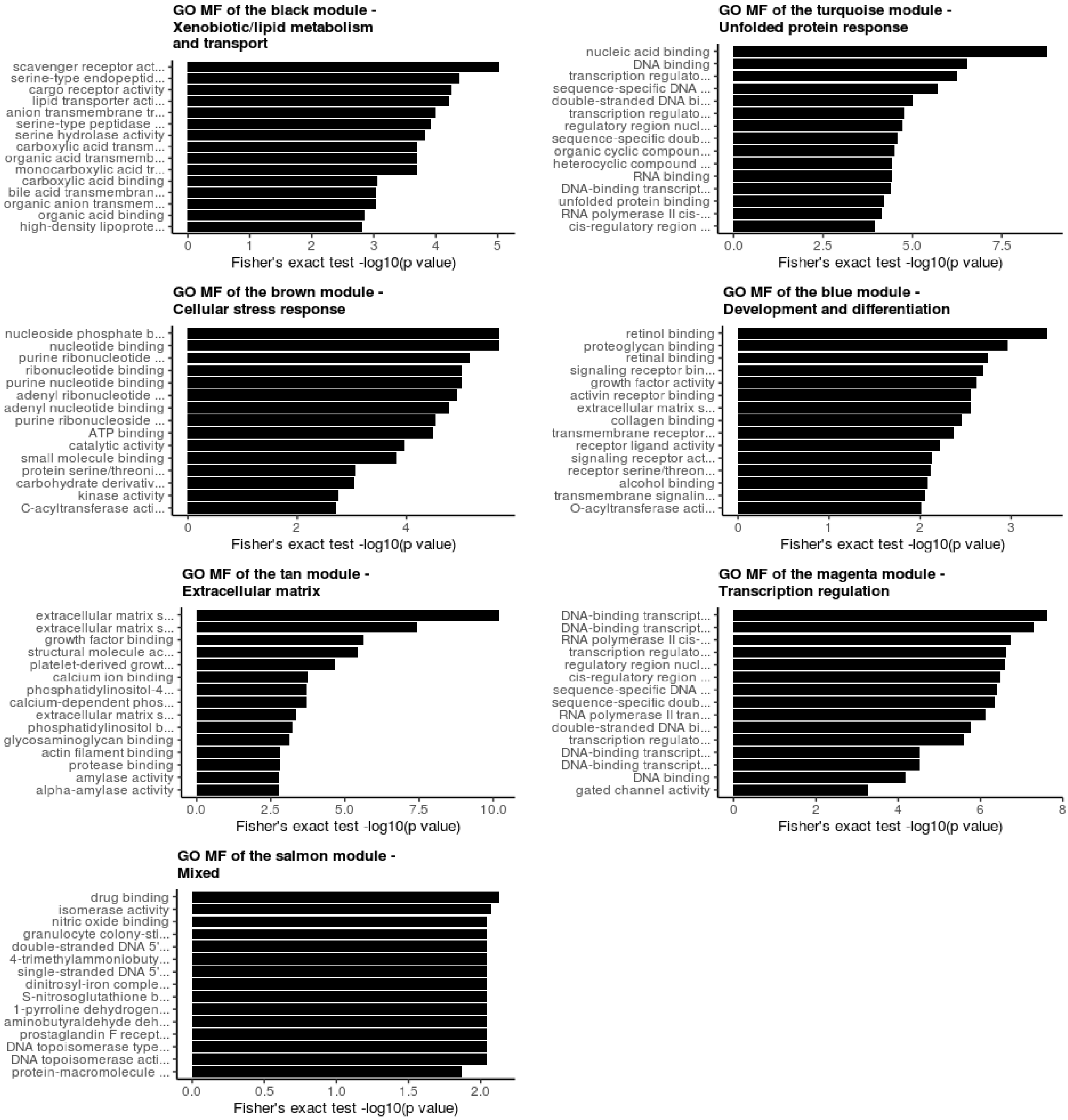
GO MF of the black module - Xenobiotic/lipid metabolism and transport
scavenger receptor act...
serine-type endopeptid...
cargo receptor activity
lipid transporter acti...
anion transmembrane tr...
serine-type peptidase ...
serine hydrolase activity
carboxylic acid transm...
organic acid transmemb...
monocarboxylic acid tr...
carboxylic acid binding
bile acid transmembran...
organic anion transmem...
organic acid binding
high-density lipoprote...
0 1 2 3 4 5
Fisher's exact test -log10(p value)
GO MF of the turquoise module - Unfolded protein response
nucleic acid binding
DNA binding
transcription regulato...
sequence-specific DNA ...
double-stranded DNA bi...
transcription regulato...
regulatory region nucl...
sequence-specific doub...
organic cyclic compoun...
heterocyclic compound ...
RNA binding
DNA-binding transcript...
unfolded protein binding
RNA polymerase II cis-...
cis-regulatory region ...
0.0 2.5 5.0 7.5
Fisher's exact test -log10(p value)
GO MF of the brown module - Cellular stress response
nucleoside phosphate b...
nucleotide binding
purine ribonucleotide ...
ribonucleotide binding
purine nucleotide binding
adenyl ribonucleotide ...
adenyl nucleotide binding
purine ribonucleoside ...
ATP binding
catalytic activity
small molecule binding
protein serine/threoni...
carbohydrate derivativ...
kinase activity
C-acyltransferase acti...
0 2 4
Fisher's exact test -log10(p value)
GO MF of the blue module - Development and differentiation
retinol binding
proteoglycan binding
retinal binding
signaling receptor bin...
growth factor activity
activin receptor binding
extracellular matrix s...
collagen binding
transmembrane receptor...
receptor ligand activity
signaling receptor act...
receptor serine/threon...
alcohol binding
transmembrane signalin...
O-acyltransferase acti...
0 1 2 3
Fisher's exact test -log10(p value)
GO MF of the tan module - Extracellular matrix
extracellular matrix s...
extracellular matrix s...
growth factor binding
structural molecule ac...
platelet-derived growt...
calcium ion binding
phosphatidylinositol-4...
calcium-dependent phos...
extracellular matrix s...
phosphatidylinositol b...
glycosaminoglycan binding
actin filament binding
protease binding
amylase activity
alpha-amylase activity
0.0 2.5 5.0 7.5 10.0
Fisher's exact test -log10(p value)
GO MF of the magenta module - Transcription regulation
DNA-binding transcript...
DNA-binding transcript...
RNA polymerase II cis-...
transcription regulato...
regulatory region nucl...
cis-regulatory region ...
sequence-specific DNA ...
sequence-specific doub...
RNA polymerase II tran...
double-stranded DNA bi...
transcription regulato...
DNA-binding transcript...
DNA-binding transcript...
DNA binding
gated channel activity
0 2 4 6 8
Fisher's exact test -log10(p value)
GO MF of the salmon module - Mixed
drug binding
isomerase activity
nitric oxide binding
granulocyte colony-sti...
double-stranded DNA 5'...
4-trimethylammoniobuty...
single-stranded DNA 5'...
dinitrosyl-iron comple...
S-nitrosoglutathione b...
1-pyrroline dehydrogen...
aminobutyraldehyde deh...
prostaglandin F recept...
DNA topoisomerase type...
DNA topoisomerase acti...
protein-macromolecule ...
0.0 0.5 1.0 1.5 2.0
Fisher's exact test -log10(p value)

a

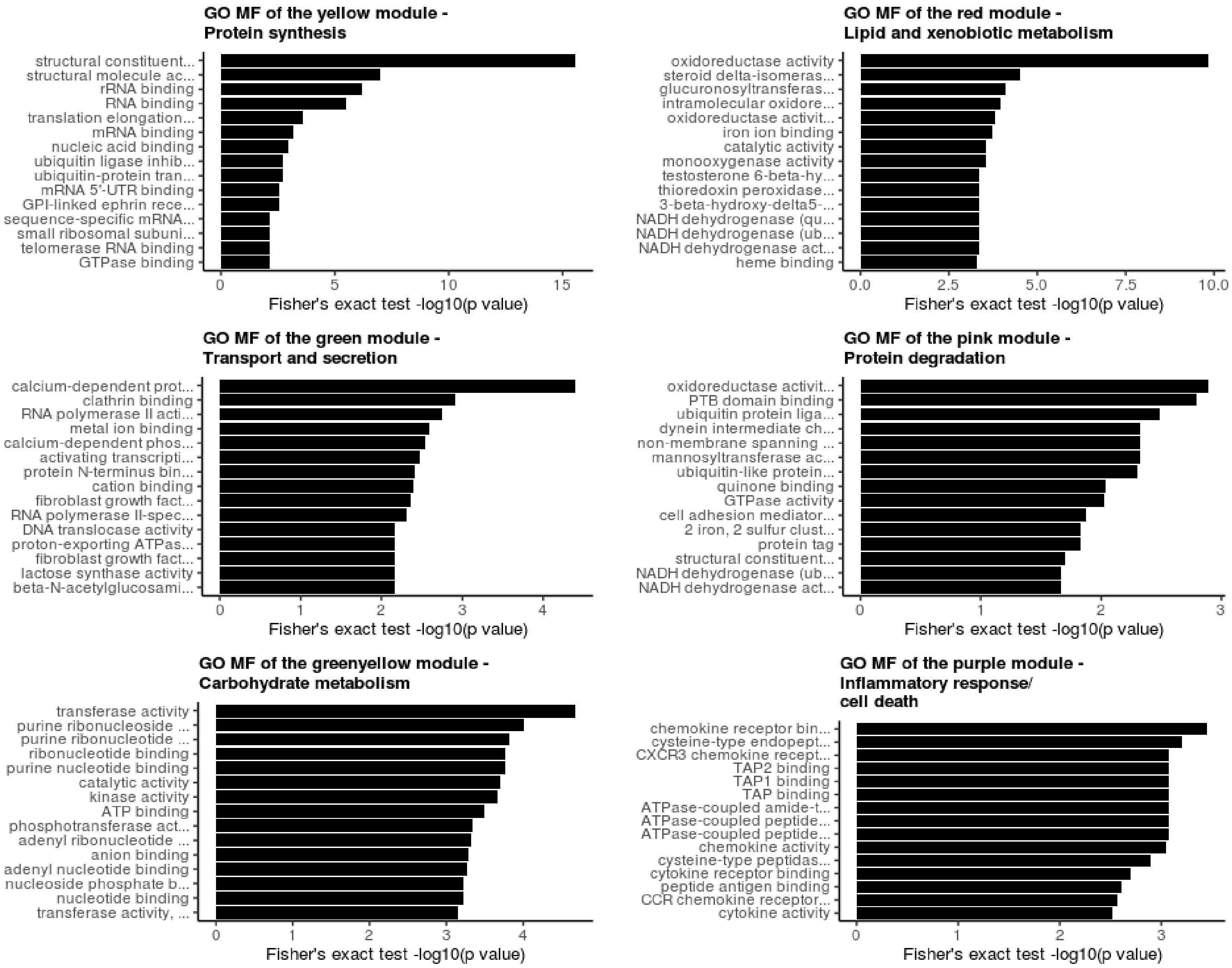

b

Figure S8. a. O Molecular Functions for clusters obtained from WGCNA (Figure 3.b.). Black -lipid and xenobiotic metabolism and transport; turquoise—Unfolded Protein Response (UPR); brown—cellular stress response; blue—development and differentiation; tan—Extracellular Matrix (ECM); magenta—transcription regulation; Salmon–mixed. **b.** O Molecular Functions for clusters obtained from WGCNA (Figure 3.b.). Yellow -protein synthesis; red—lipid and xenobiotic metabolism; green—transport and secretion; pink—protein degradation; greenyellow—carbohydrate metabolism; purple—inflammatory response/cell death.

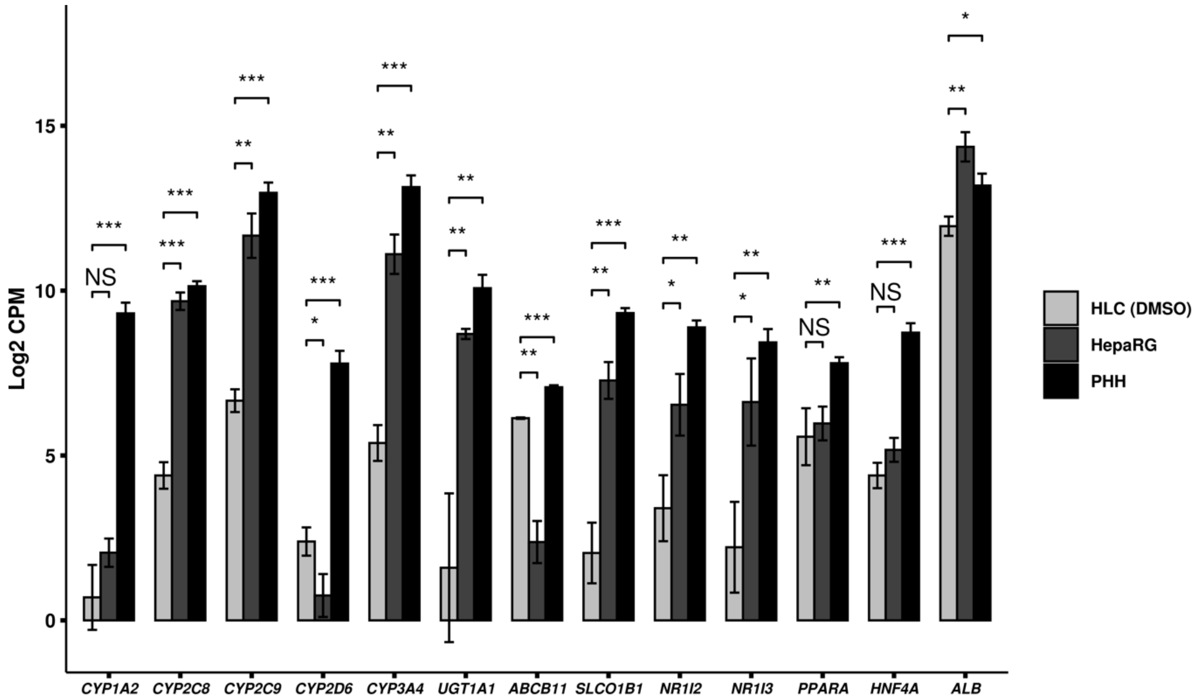

Figure S9. Expression levels of genes involved in phase I and phase II metabolism and mature hepatocyte markers from TempO-Seq data, in in SBAD2-3x-AAGLY-HLCs relative to PHHs and HepaRG cells. (*: $p < 0.05$, ** : $p < 0.01$, *** : $p < 0.001$, NS: Not significant).

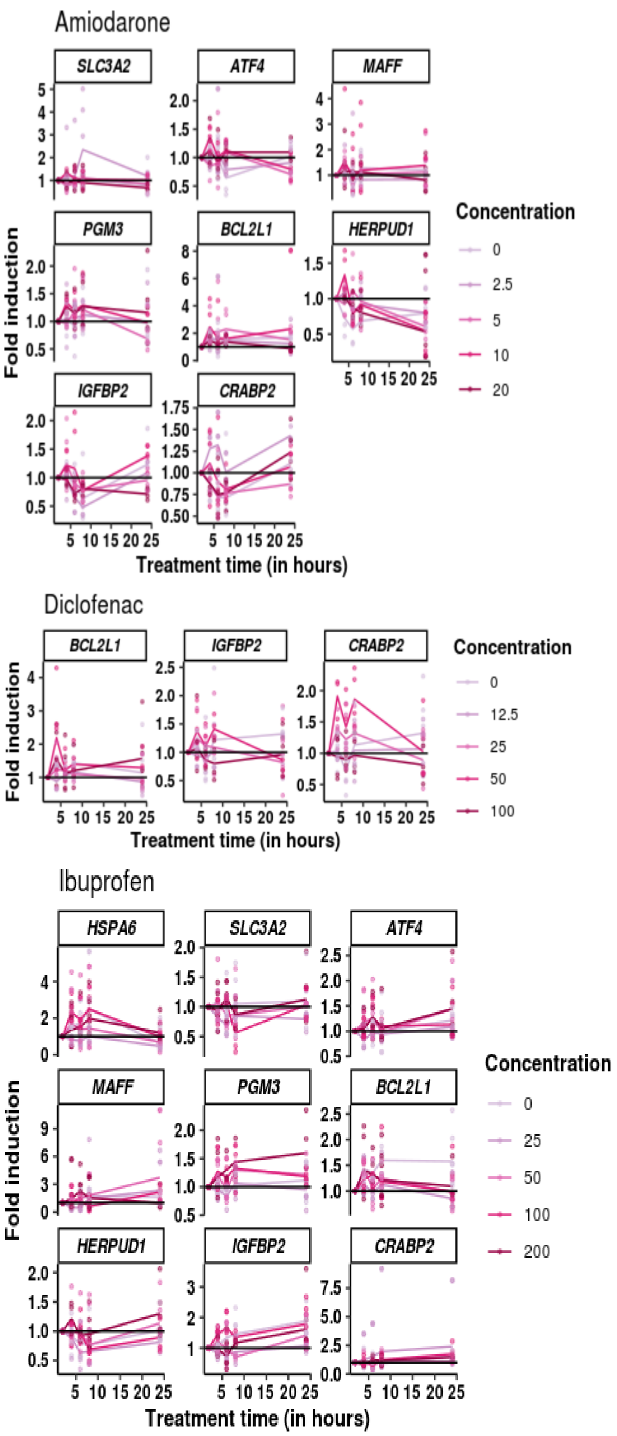

Figure S10. Cellular stress genes show differential expression at different time-points after treatment of SBAD2-3x-AAGLY HLCs with Amiodarone, Diclofenac, and Ibuprofen. RT-qPCR analysis of the genes selected in d40 SBAD2-3x-AAGLY HLCs treated with increasing concentrations of amiodarone, diclofenac, or ibuprofen over a period of 24 h, for genes as identified in Figure 4.a. X-axes indicate the treatment times.

References:

1. Bock, C.; Kiskinis, E.; Verstappen, G.; Gu, H.; Boulting, G.; Smith, Z.D.; Ziller, M.; Croft, G.; Amoroso, M.W.; Oakley, D.; et al. Reference Maps of Human ES and iPS Cell Variation Enable High-Throughput Characterization of Pluripotent Cell Lines. Cell 2011, 144, 439–452. https://doi.org/10.1016/j.cell.2010.12.032.

2. Xu, J.J.; Henstock, P.V.; Dunn, M.C.; Smith, A.R.; Chabot, J.R.; De Graaf, D. Cellular Imaging Predictions of Clinical Drug-Induced Liver Injury. *Toxicol. Sci.* **2008**, *105*, 97–105. https://doi.org/10.1093/toxsci/kfn109.
3. Lin, W.; Huang, Y.-W.; Zhou, X.-D.; Ma, Y. Toxicity of Cerium Oxide Nanoparticles in Human Lung Cancer Cells. *Int. J. Toxicol.* **2006**, *25*, 451–457. https://doi.org/10.1080/10915810600959543.
4. García-Rubio, L.; García-Abad, A.M.; Soler, F.; Míguez, M.P. Cytotoxicity of paraquat in freshly isolated rat hepatocytes: Effects of L-carnitine. *BioFactors* **1998**, *8*, 59–64. https://doi.org/10.1002/biof.5520080111.
5. Bramlage, P.; Goldis, A. Bioequivalence study of three ibuprofen formulations after single dose administration in healthy volunteers. *BMC Pharmacol.* **2008**, *8*, 18. https://doi.org/10.1186/1471-2210-8-18.
6. Barpe, D.R.; Rosa, D.D.; Froehlich, P.E. Pharmacokinetic evaluation of doxorubicin plasma levels in normal and overweight patients with breast cancer and simulation of dose adjustment by different indexes of body mass. *Eur. J. Pharm. Sci.* **2010**, *41*, 458–463. https://doi.org/10.1016/j.ejps.2010.07.015.
7. Tchounwou, P.B.; Yedjou, C.G.; Foxx, D.N.; Ishaque, A.B.; Shen, E. Lead-induced cytotoxicity and transcriptional activation of stress genes in human liver carcinoma (HepG2) cells. *Mol. Cell. Biochem.* **2004**, *255*, 161–170. https://doi.org/10.1023/b:mcbi.0000007272.46923.12.
8. Regec, A.L.; Trifillis, A.L.; Trump, B.F. The Effect of Gentamicin on Human Renal Proximal Tubular Cells. *Toxicol. Pathol.* **1986**, *14*, 238–241. https://doi.org/10.1177/019262338601400213.
9. Rha, J.H.; Jang, I.J.; Lee, K.H.; Chong, W.S.; Shin, S.G.; Lee, N.; Myung, H.J. Pharmacokinetic comparison of two valproic acid formulations: A plain and a controlled release enteric-coated tablets. *J. Korean Med. Sci.* **1993**, *8*, 251–256. https://doi.org/10.3346/jkms.1993.8.4.251.
10. Proctor, W.R.; Foster, A.J.; Vogt, J.; Summers, C.; Middleton, B.; Pilling, M.; Shienson, D.; Kijanska, M.; Ströbel, S.; Kelm, J.M.; et al. Utility of spherical human liver microtissues for prediction of clinical drug-induced liver injury. *Arch. Toxicol.* **2017**, *91*, 2849–2863. https://doi.org/10.1007/s00204-017-2002-1.
11. Tsutsumi, S.; Gotoh, T.; Tomisato, W.; Mima, S.; Hoshino, T.; Hwang, H.-J.; Takenaka, H.; Tsuchiya, T.; Mori, M.; Mizushima, T. Endoplasmic reticulum stress response is involved in nonsteroidal anti-inflammatory drug-induced apoptosis. *Cell Death Differ.* **2004**, *11*, 1009–1016. https://doi.org/10.1038/sj.cdd.4401436.
12. Wong, W.W.-L.; Puthalakath, H. Bcl-2 family proteins: The sentinels of the mitochondrial apoptosis pathway. *IUBMB Life* **2008**, *60*, 390–397. https://doi.org/10.1002/iub.51.
13. Hori, O.; Ichinoda, F.; Yamaguchi, A.; Tamatani, T.; Taniguchi, M.; Koyama, Y.; Katayama, T.; Tohyama, M.; Stern, D.M.; Ozawa, K.; et al. Role of Herp in the endoplasmic reticulum stress response. *Genes Cells* **2004**, *9*, 457–469. https://doi.org/10.1111/j.1356-9597.2004.00735.x.
14. Katsuoka, F.; Motohashi, H.; Ishii, T.; Aburatani, H.; Engel, J.D.; Yamamoto, M. Genetic Evidence that Small Maf Proteins Are Essential for the Activation of Antioxidant Response Element-Dependent Genes. *Mol. Cell. Biol.* **2005**, *25*, 8044–8051. https://doi.org/10.1128/mcb.25.18.8044-8051.2005.
15. Liu, C.; Li, X.; Li, C.; Zhang, Z.; Gao, X.; Jia, Z.; Chen, H.; Jia, Q.; Zhao, X.; Liu, J.; et al. SLC3A2 is a novel endoplasmic reticulum stress-related signaling protein that regulates the unfolded protein response and apoptosis. *PLoS ONE* **2018**, *13*, e0208993. https://doi.org/10.1371/journal.pone.0208993.
16. Ashida, H.; Kanazawa, K.; Danno, G.-I. Hepatic Phosphoglucomutase Activity as a Marker of Oxidative Stress Induced by Pro-oxidative Drugs. *Biosci. Biotechnol. Biochem.* **1994**, *58*, 55–59. https://doi.org/10.1271/bbb.58.55.
17. Ordovás, L.; Boon, R.; Pistoni, M.; Chen, Y.; Wolfs, E.; Guo, W.; Sambathkumar, R.; Bobis-Wozowicz, S.; Helsen, N.; Vanhove, J.; et al. Efficient Recombinase-Mediated Cassette Exchange in hPSCs to Study the Hepatocyte Lineage Reveals AAVS1 Locus-Mediated Transgene Inhibition. *Stem Cell Rep.* **2015**, *5*, 918–931. https://doi.org/10.1016/j.stemcr.2015.09.004.
